# Performance, combustion, emission and optimization characteristics of biodiesel–n-butanol blends enriched with Ni_2_O_3_ nanoparticles in a diesel engine

**DOI:** 10.1038/s41598-026-36115-y

**Published:** 2026-01-17

**Authors:** Ali Serkan Avcı, Seda Fahriye Yavaşoğlu

**Affiliations:** 1 Besiri OSB Vocational School, Batman University, 72100 Batman, Turkey; 2 Turkish Petroleum Corporation, 72100 Batman, Turkey

**Keywords:** Engine performance, Combustion, Exhaust emissions, Optimization, Ni_2_O_3_, Energy science and technology, Engineering, Environmental sciences

## Abstract

In the pursuit of sustainable and clean energy, biofuels and nanofuels are increasingly investigated as practical solutions to improve diesel engine efficiency and emission characteristics. This study evaluates the effects of Nickel (III) oxide nanoparticles added to biodiesel (B20) and biodiesel–n-butanol (B20But10) blends on combustion, engine performance, emissions, and optimization using a single-cylinder, four-stroke, water-cooled, direct injection diesel engine. Experiments were conducted at multiple engine loads and nanoparticle concentrations ranging from 0 to 100 ppm. At full load and 100 ppm Ni₂O₃, peak in-cylinder pressure increased to 55.86 bar for B20 and 55.45 bar for B20But10, while maximum heat release rates reached 29.45 and 30.02 J/°CA, indicating enhanced premixed combustion behavior. Brake thermal efficiency increased to 24.89% for B20 and 24.94% for B20But10, accompanied by reductions in brake specific fuel consumption to 0.309 and 0.333 kg/kWh, respectively. Emission results showed reductions of 13–28% in HC, 8–43% in smoke opacity, and 12–21% in NO_x_. Response surface methodology was employed to develop both performance- and emission-oriented predictive models, yielding high reliability (R^2^ = 90.9–99.9%) and identifying optimal nanoparticle levels between 50 and 75 ppm. Overall, Ni₂O₃-enhanced blends provide measurable performance and emission benefits without requiring engine modifications.

## Introduction

Despite the rapid growth of renewable energy technologies, the global energy system still relies heavily on fossil fuels, which account for approximately 81.5% of primary energy supply^1^. This dependency has led to a significant increase in greenhouse gas emissions, particularly from the transportation sector. The transportation sector is responsible for about 15% of total anthropogenic greenhouse gas emissions and 23% of energy-related CO₂ emissions; moreover, it has been the fastest-growing end use sector in terms of emission increases over the past decade. Rapid urbanization and the rising number of motor vehicles have further deteriorated air quality, especially in densely populated urban areas that account for nearly 70% of global pollutant emissions, thereby placing additional pressure on infrastructure^2^. In response, the European Green Deal (with the target of achieving climate neutrality by 2050 and at least a 71% reduction in emissions by 2030) and the United Nations Sustainable Development Goals (particularly SDG 7: Affordable and Clean Energy and SDG 13: Climate Action) emphasize the urgency of reducing dependence on fossil fuels in transportation^3,4^. In this context, renewable alternatives such as biodiesel and alcohol-based fuels are increasingly considered as strategic solutions for low-emission transportation applications.

Due to their favorable power to weight ratio and ability to respond quickly to load changes, internal combustion engines remain widely used in both transportation and industrial applications. Recent research has increasingly aimed to enhance their combustion performance, thermal efficiency, and emission profiles by adopting strategies such as fuel reformulation, injection parameter optimization, and advanced combustion techniques^5,6^. In line with the global trend toward cleaner and more sustainable energy systems, alternative fuels for compression ignition (CI) engines have received growing attention.

Biodiesel, an oxygenated renewable fuel produced from vegetable oils or animal fats through transesterification, is one of the most extensively studied alternatives to petrodiesel^7^. Its compatibility with existing diesel engines, high cetane number, good lubricity, and biodegradable nature make biodiesel an attractive option for sustainable transportation. In the literature, it has been reported that blending biodiesel with diesel fuel significantly reduces brake specific fuel consumption (BSFC) along with HC, CO, and CO₂ emissions^8,9^. However, due to its lower energy content, slight variations in brake thermal efficiency (BTE) have been observed^10,11^. Furthermore, the combustion duration and flame propagation may vary depending on the type of biodiesel. In addition, the higher viscosity and density of biodiesel can lead to insufficient atomization, higher fuel consumption, and cold-start problems^12,13^. These drawbacks are further exacerbated by its low volatility and poor cold-flow properties, which may cause crystal formation and solidification of fatty acids at low temperatures. Such limitations can be mitigated by blending with short and medium chain alcohols (e.g., ethanol, methanol, butanol), which possess higher oxygen content and lower viscosity.

n-Butanol (C₄H₉OH), a four-carbon linear alcohol, is a higher-chain alcohol that can be produced from biomass via fermentation and can be utilized as a fuel additive in both compression ignition (CI) and spark ignition (SI) engines^14^. Compared to lower-chain alcohols such as ethanol and methanol, its higher molecular weight provides better miscibility with diesel fuel, higher energy content, and more stable blending characteristics^15^. When blended with diesel, it does not cause phase separation; moreover, its higher viscosity and density enhance lubricating properties while exerting less corrosive effects on engine components^16^. The low vapor pressure, high cetane number, and high boiling point of n-butanol balance the evaporation rate after injection, facilitate the formation of a more homogeneous fuel–air mixture, and ensure a more controlled combustion process^17^. In addition, its high flash point reduces safety requirements during fuel storage and transportation^18^. Due to its physicochemical properties, oxygen content, and chain length, n-butanol has the potential to improve both the premixed combustion phase and the diffusion combustion phase^19^. The literature reports that the addition of n-butanol enhances combustion efficiency and reduces harmful emissions; however, it has also been reported to increase fuel consumption^20–22^. When blended with biodiesel, n-butanol mitigates atomization problems caused by the high viscosity of biodiesel, improves evaporation characteristics, and enhances combustion quality through its oxygen content. This synergistic effect is particularly evident in the reduction of CO, HC, and smoke emissions^23^. Accordingly, recent studies have increasingly explored the role of additives, particularly nanoparticles (NPs), in further enhancing the performance and emission characteristics of biodiesel–n-butanol blends.

While numerous studies have investigated biodiesel and NP-enhanced fuels, most focus on neat biodiesel substitution or isolated additive effects. In contrast, the present study adopts a blended-fuel strategy (B20 and B20But10) to reflect realistic and currently applicable fuel utilization scenarios rather than unrealistic full diesel replacement. The biodiesel employed was selected for its high quality and compliance with EN 14214 standards, characterized by a high cetane number, superior flash point, and high ester content, which ensure favorable ignition and safety in compression-ignition engines. It is widely recognized that infrastructure and economic constraints limit the immediate full-scale replacement of petroleum diesel. However, low-to-mid-level blends like B20 are already implemented as vital transitional fuels. Accordingly, the objective of this work is not to promote complete substitution but to enhance the combustion and emission profiles of these practically relevant blends through the synergistic addition of n-butanol and Ni₂O₃ NPs without requiring engine modification.

In recent years, NPs have emerged as promising additives to overcome the performance and emission limitations of alternative fuels. Due to their small size and high surface to volume ratio, these particles enhance fuel atomization and promote a more efficient combustion process^24,25^. NPs can generally be classified into metallic and metal oxide categories. Among them, metal oxide-based NPs represent one of the most advantageous groups as fuel additives in alternative fuels. With their high catalytic activity, oxygen-carrying capacity, and thermal conductivity, these NPs play an effective role in reducing ignition delay, improving combustion efficiency, and lowering harmful emissions in the combustion chamber^26–28^. The most commonly used metal oxide NPs reported in the literature include aluminum oxide^29^, copper oxide^30^, iron oxide^31^, zinc oxide^32^, manganese oxide^33^, cerium oxide^34^, and nickel oxide^35^. Acting as oxidation catalysts, these additives promote more complete and cleaner combustion of the fuel. To achieve a more holistic understanding of how NPs influence ecological balance, future studies should address their long-term impacts, examine interactions among various NP types, and explore potential indirect effects that may propagate through food webs^36^.

Nickel-based NPs are among the most prominent additives in alternative fuel technologies due to their superior catalytic and thermophysical properties^37^. In the literature, Nickel (III) oxide (Ni₂O₃) has been reported to catalyze oxidation reactions during combustion owing to its high surface area, thermal conductivity, and oxygen storage/transport capacity^38,39^. These features have the potential to facilitate the formation of a more homogeneous air–fuel mixture inside the cylinder, reduce ignition delay, increase flame propagation speed, and optimize combustion temperature^40–42^. Several studies have demonstrated that Ni₂O₃ can accelerate catalytic oxidation, enabling more complete combustion at lower temperatures, while their high thermal conductivity enhances heat transfer and promotes secondary atomization by reducing fuel droplet size^43,44^. This mechanism may alleviate the atomization issues associated with the relatively high viscosity of biodiesel and create a synergistic effect with low viscosity but high latent heat components such as n-butanol, thereby improving combustion efficiency. Although findings on the dispersion behavior of nickel oxide NPs in the literature are limited, several studies have indicated that different metal oxide NPs can form short-term stable suspensions in similar fuel environments^45,46^. This provides a theoretical basis for the assumption that Ni₂O₃ can also be homogeneously dispersed in polar fuel blends such as biodiesel–n-butanol. Furthermore, its high thermal conductivity is reported to balance the in-cylinder temperature distribution, limit the formation of locally rich mixture zones, and contribute to maintaining catalytic activity throughout engine operation^47,48^. Therefore, the selection of Ni₂O₃ as a fuel additive in this study is considered a rational choice, supported by its catalytic and thermophysical properties reported in the literature, its potential dispersion behavior, and the theoretical and experimental evidence of its positive effects on combustion efficiency and exhaust emissions.

The studies summarized in Table 1 demonstrate that nickel-based NP additives can improve combustion and emission performance in diesel engines. Ni and NiO additives accelerate in-cylinder oxidation reactions, thereby reducing CO, HC, and smoke emissions; when blended with oxygen-rich fuels such as biodiesel and n-butanol, they further enhance mixture homogeneity and improve flame propagation. However, despite their promising potential as fuel additives, Table 1 shows that Ni or NiO additives have mostly been evaluated with diesel and biodiesel fuels in terms of engine performance and emissions, while combustion characteristics such as in-cylinder pressure (CP), heat release rate (HRR), cumulative heat release rate (CHRR), pressure rise rate (PRR), and ignition delay (ID), as well as multi-objective optimization scenarios and economic analyses, have not been comprehensively addressed. Considering the European Union’s Green Deal target of a 100% reduction in vehicle emissions by 2035 and the upcoming EURO 7 standards in 2025^49^, the aim of this study is to enrich biodiesel–n-butanol blends with Ni₂O₃ and to develop a fuel that can contribute to carbon–neutral goals through a holistic analysis without requiring engine modifications.

**Table 1:** Effects of metal oxide NP additives on diesel engine combustion, engine performance and emissions in the literature.

Fuel blend	NP	Engine type and test condition	Combustion performance	Engine performance	Emission characteristics	Ref
B20But30 (diesel-biodiesel–n-butanol)	ZnO	Single-cylinder, four-stroke, DI, Kirloskar TV1, 5.2 kW, 1500 rpm	↑EGT	↑BTE, ↓BSFC	↓CO, ↑HC, ↓NOx, ↑CO₂	^50^
B100E5B5 (biodiesel-etanol-n-butanol)	Fe₃O₄	Single-cylinder, four-stroke, air-cooled, Yanmar L70N, CR 20:1	↓ID	↑BTE, ↓BSFC	↓CO, ↓HC, ↓Smoke, ↓NOx	^51^
D70B20E10 (diesel-etanol-n-butanol)	Al₂O₃	Single-cylinder, four-stroke, DI diesel engine, 4.4 kW, 1500 rpm	↑CP, ↑HRR, ↑CHRR, ↓ID	↑BTE, ↓BSFC	↓CO, ↓HC, ↓NOx, ↓Smoke	^52^
D80B10But10 Diesel-biodiesel–n-butanol	Ni	Single-cylinder, four-stroke, air-cooled, DI, 5.4 kW, 3600 rpm	-	↑BTE, ↓BSFC	↓CO, ↓NOx, ↑CO₂	^40^
B20 (mahua methyl ester- diesel)	NiO	Single-cylinder, four-stroke, DI, water-cooled, VCR, 4.2 kW,1500 rpm	-	↑BTE, ↓BSFC	↓CO, ↓HC, ↓NOx	^41^
NBE25 (azadirachta indica-diesel)	NiO	Single-cylinder, DI-CI, water-cooled, VCR engine, 4.8 kW, 1500 rpm	-	↑BTE, ↓BSFC	↓CO, ↓HC, ↑CO₂, ↑NOx	^42^
TE60 (tall oil methyl ester-diesel)	NiO	Single-cylinder, four-stroke, air-cooled, DI, 6.3 kW, 3600 rpm	-	↓BSFC	↓CO, ↓Smoke, ↓NOx	^53^
B15 (n-pentane-diesel)	NiO₂	Single-cylinder, four-stroke, water-cooled, DI,3.5 kW,1475 rpm	↑EGT	↑BTE, ↓BSFC	↓CO, ↓HC, ↓NOx, ↓Smoke, ↑CO₂	^54^
B20 (coconut oil-diesel)	NiFe₂O₄	Six-cylinder, turbocharged, CRDI diesel engine, 1500 rpm	-	↑BTE, ↓BSFC	↓CO, ↓HC, ↑CO₂, ↓NOx	^55^
Diesel	NiFe₂O₄	Six-cylinder, four-stroke, turbocharged, intercooled, CRDI, 184 kW, 2500 rpm	-	-	↓CO, ↓HC, ↓NOx	^56^
Isodiesel (diesel-biodiesel)	NiFe₂O₄	Single-cylinder, four-stroke, direct injection, 4.4 kW, 1500 rpm	-	-	↓CO, ↓HC, ↓NOx, ↓Smoke	^57^

In studies on engine performance and emission characteristics, response surface methodology (RSM) is widely preferred due to its ability to reliably model the complex interactions of multiple parameters and its success in determining optimal conditions^58,59^. In the literature, the reduction of experimental runs, along with time and cost advantages, as well as its capacity to provide statistically robust predictions, are cited as the main reasons for researchers’ preference for RSM^60–62^. In this study, RSM was employed to comprehensively and optimizable analyze the effects of NP concentration and engine load variables on engine characteristics.

## Aim of the study

The main objectives of this study are summarized as follows:

- This study aims to establish a technically robust and application-oriented framework for Ni₂O₃ NP–enriched biodiesel-based fuel blends by integrating morphological characterization, detailed combustion analysis, engine performance metrics, exhaust emission characteristics, and statistically based multi-objective optimization.
- After confirming the suitability of Ni₂O₃ NPs as fuel additives through morphological characterization, the effects of Ni₂O₃ addition at different concentrations (25, 50, 75, and 100 ppm) were systematically investigated under varying engine loads (0.3, 1.0, 2.0, and 3.0 BMEP) using a single-cylinder direct-injection diesel engine fueled with B20 and B20But10 blends.
- While previous studies on nickel-based NP-enhanced diesel fuels have largely remained descriptive and have reported performance and emission trends at discrete operating points without offering a transferable optimization framework for conflicting objectives, this study adopts a quantitative modeling approach. RSM/CCD-based response surface models were developed to quantitatively characterize the coupled influence of Ni₂O₃ dosage and BMEP on engine performance and emissions by explicitly incorporating interaction and quadratic effects, and the resulting models were subsequently integrated into a multi-objective optimization framework.
- Five independent optimization scenarios were formulated to represent different decision-making perspectives, including performance-oriented, emission-oriented, balanced performance–emission, and priority-weighted strategies. Through desirability-based optimization, the study moves beyond identifying a single optimal operating point and instead defines a practically meaningful and robust optimal operating region.
- The economic feasibility of Ni₂O₃-doped fuel blends was systematically evaluated alongside their technical performance to provide a realistic and application-oriented assessment.
- By addressing existing gaps in the literature, where most nickel-based NP studies primarily focus on performance and emission outcomes, the present study extends this scope by explicitly incorporating detailed combustion characteristics (CP, HRR, CHRR, PRR, and ID), together with response surface–based modeling, into an environmentally and performance-oriented multi-objective optimization framework.

## Materials and methods

### Properties of Ni₂O₃

In this study, Ni₂O₃, to be used for the first time in the literature as a fuel additive, were supplied from Nanografi Co. (Ankara, Turkey). The technical properties of the material are presented in Table 2. Ni₂O₃ are widely utilized not only in biological applications but also to enhance the electrical conductivity, magnetic behavior, and optical properties of composite materials^63^.

**Table 2:** Details of Ni₂O₃ NP.

Name	Values
Compound	Ni_2_O_3_
Producer	Nanografi nanotechnology
Product number	NG01NM1802
Purity (%)	99.95 +
Color	Black-dark gray
Average particle size (nm)	40
Specific Surface Area (m^2^/g)	40–60
Bulk density (g/cm3)	0.38
True density (g/cm3)	4.84
Elemental analysis (wt %)	Ni_2_O_3_CoPbFeCuCa 99.95 < 0.0140.010.0280.010.035

**Fig. 1:**
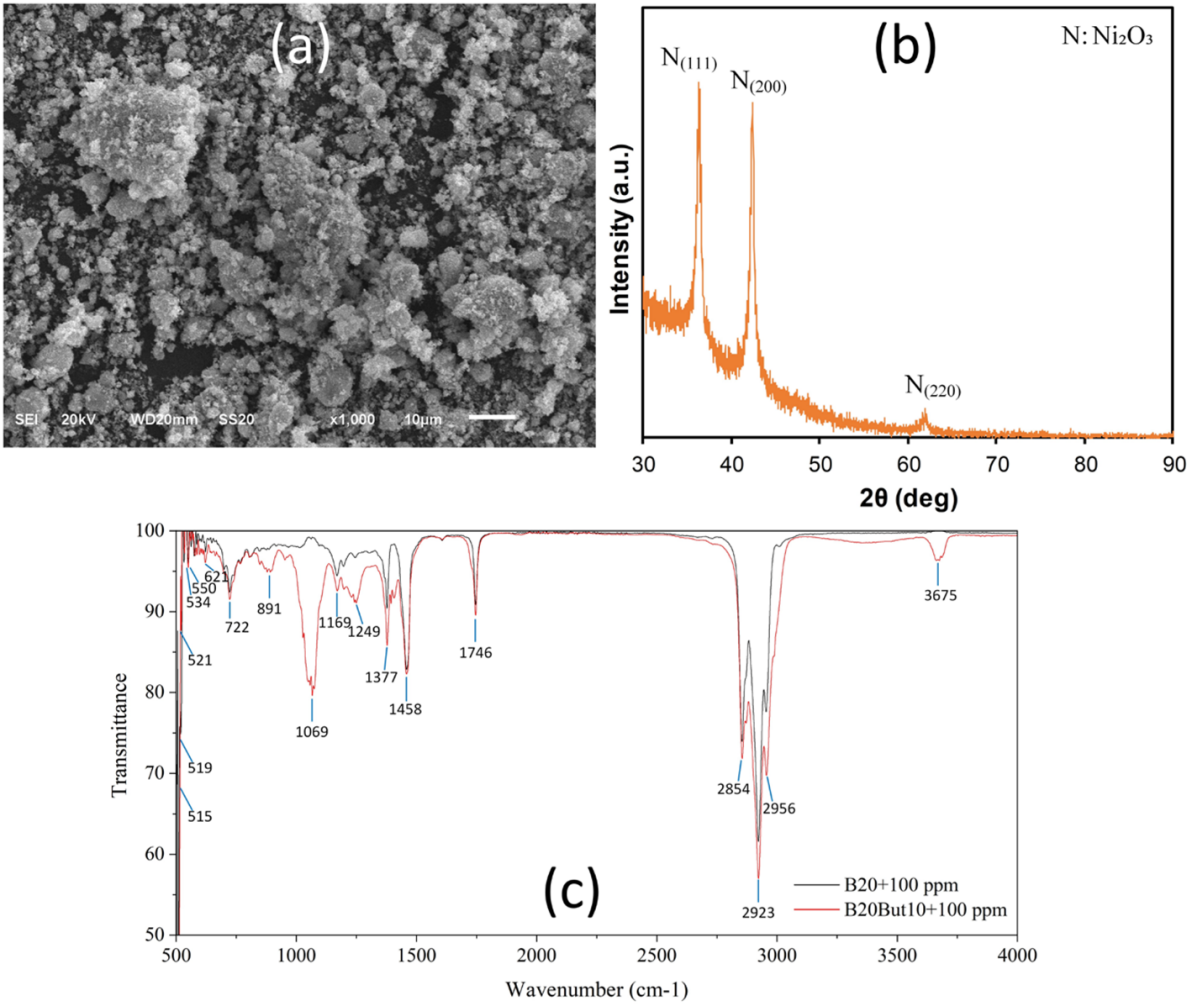
Ni₂O₃ NP (**a**) SEM image, (**b**) XRD pattern (**c**) FTIR spectra of B20 and B20But10 fuels blended with 100 ppm Ni₂O₃.

In Fig. 1, the morphology of Ni₂O₃, along with structural parameters such as crystal lattice, phase structure, and particle size, was evaluated using SEM, XRD, and FTIR analyses. Figure 1a shows the surface morphology of the synthesized Ni₂O₃. The images reveal that the particles are predominantly spherical or semi-spherical in shape and exhibit a pronounced tendency toward agglomeration due to their high surface energies. The average particle sizes fall within the nanoscale range; however, microsphere-like clusters were also observed in some regions. These irregular agglomerations are considered to increase the overall surface area of the material, which can provide advantages in catalytic and adsorptive applications^45^. In addition, the rough surface morphology may contribute to a higher number of active sites, enabling more effective dispersion of the NPs within the fuel^64^.

Figure 1b presents the X-ray diffraction (XRD) patterns of Ni₂O₃. Distinct diffraction peaks were observed at respectively. In addition, weaker peaks around 62.9° and 75.4° confirm the (220) planes of Ni₂O₃^45,54^. The obtained diffraction patterns are consistent with JCPDS card number 47–1049, indicating that the synthesized particles possess a cubic phase structure^39^. The sharpness of the peaks suggests a high degree of crystallinity.

Figure 1c shows the FTIR spectra of B20 and B20But10 blends with Ni₂O₃. The broad O–H stretching band around 3675 cm⁻^1^ is more pronounced in B20But10, mainly due to the presence of free n-butanol. The aliphatic C–H stretching bands at 2854–2956 cm⁻^1^ confirm the hydrocarbon chains of the fuel. The strong ester carbonyl (C = O) peak at 1746 cm⁻^1^ verifies the FAME structure, further supported by the CH₂ bending at 1458 cm⁻^1^ and the CH₃ symmetric deformation at 1377 cm⁻^1^. Distinct C–O stretching bands at 1249–1169–1069 cm⁻^1^ indicate alcohol/ester functionalities originating from methyl esters and n-butanol^65^. At lower wavenumbers, the 621–515 cm⁻^1^ bands are attributed to metal–oxygen lattice vibrations (Ni–O, Ni–O–Ni), consistent with Ni–O stretching reported for Ni(II/III) oxides, thereby confirming the presence of Ni₂O₃ in the fuel^66^. The slight intensity increase and peak shifts observed in the B20But10 spectrum within this region may be associated with interactions of alcohol (O–H/C–O) groups with Ni-oxide surfaces through hydrogen bonding or Lewis acid–base mechanisms. Overall, the spectra verify both the fundamental fuel components and the successful incorporation of Ni₂O₃ into the blends.

To evaluate the colloidal stability of the NP-enhanced fuel blends, zeta potential measurements were considered as a quantitative indicator of NP surface charge characteristics and dispersion tendency^67^. For the B20 + Ni₂O₃ blend at a concentration of 100 ppm, the zeta potential values were in the approximate range of − 28 to − 38 mV, while slightly more negative values, ranging from − 30 to − 40 mV, were associated with the B20But10 + Ni₂O₃ blend at the same concentration. These values indicate a strong electrostatic repulsion among Ni₂O₃ NPs, which reduces agglomeration propensity and promotes dispersion stability.

The observed zeta potential behavior is consistent with the sedimentation test results, where both nanofuels remained stable over short and intermediate storage durations. Although partial settling was detected after extended storage (6 weeks), the combined zeta potential characteristics and sedimentation observations confirm that the prepared nanofuels possess sufficient colloidal stability for practical engine operation and experimental evaluation.

### Test fuels preparation

The experimental fuel samples were meticulously prepared in the workshop facilities of the Automotive Engineering Department at Batman University. In this study, B20 and B20But10 blends with Ni₂O₃ additives were prepared at different concentrations of 0, 25, 50, 75, and 100 ppm to enable a comparative investigation of exhaust emissions, combustion, and performance characteristics. In this context, B20 refers to a binary fuel blend consisting of 80% diesel and 20% biodiesel, while B20But10 is a ternary blend containing 70% diesel, 20% biodiesel, and 10% n-butanol.

The n-butanol content in the ternary blend was fixed at 10% by volume based on previous studies reporting that low to moderate butanol fractions (5–10%) improve oxygen availability and combustion quality while maintaining fuel miscibility and acceptable ignition characteristics in compression ignition engines^68^. Higher butanol ratios may lead to excessive ignition delay and reduced energy density, adversely affecting engine performance and stability^17,69^. Therefore, a 10% n-butanol fraction was selected as a practical compromise between combustion improvement and engine operability.

**Fig. 2:**
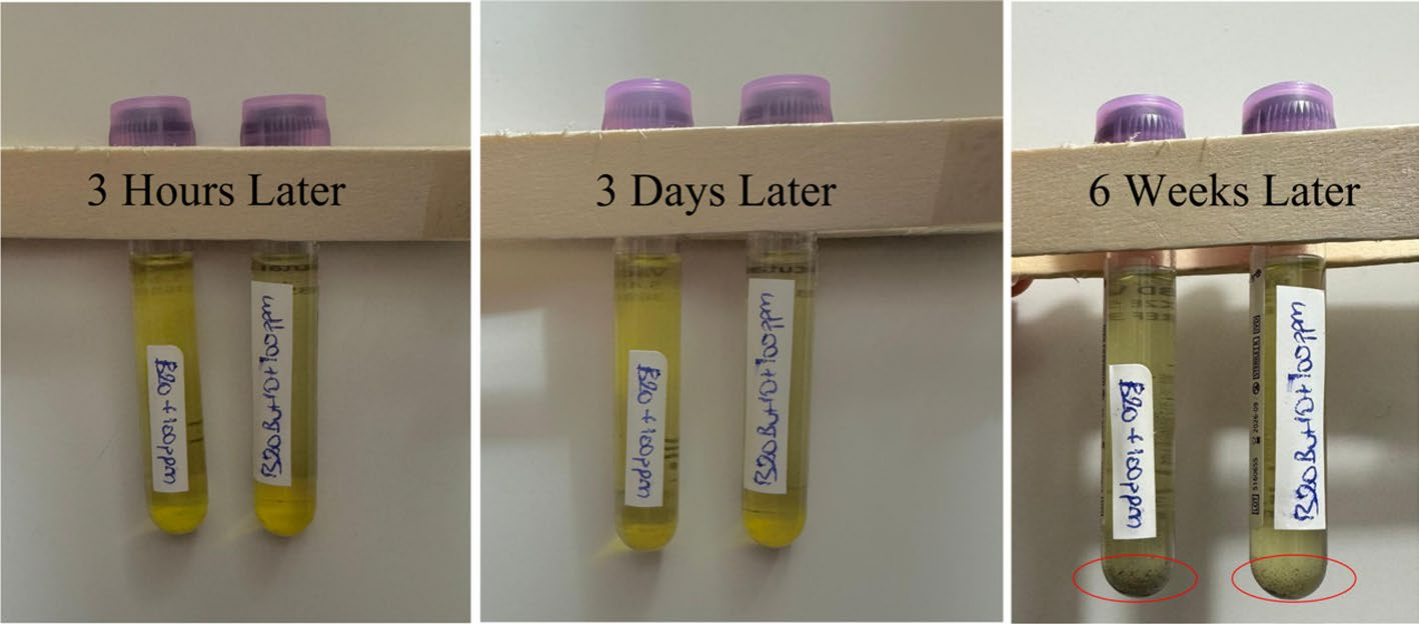
Visual sedimentation assessment of B20 and B20But10 nanofuels containing 100 ppm Ni₂O₃ during short-, mid-, and long-term storage.

**Fig. 3:**
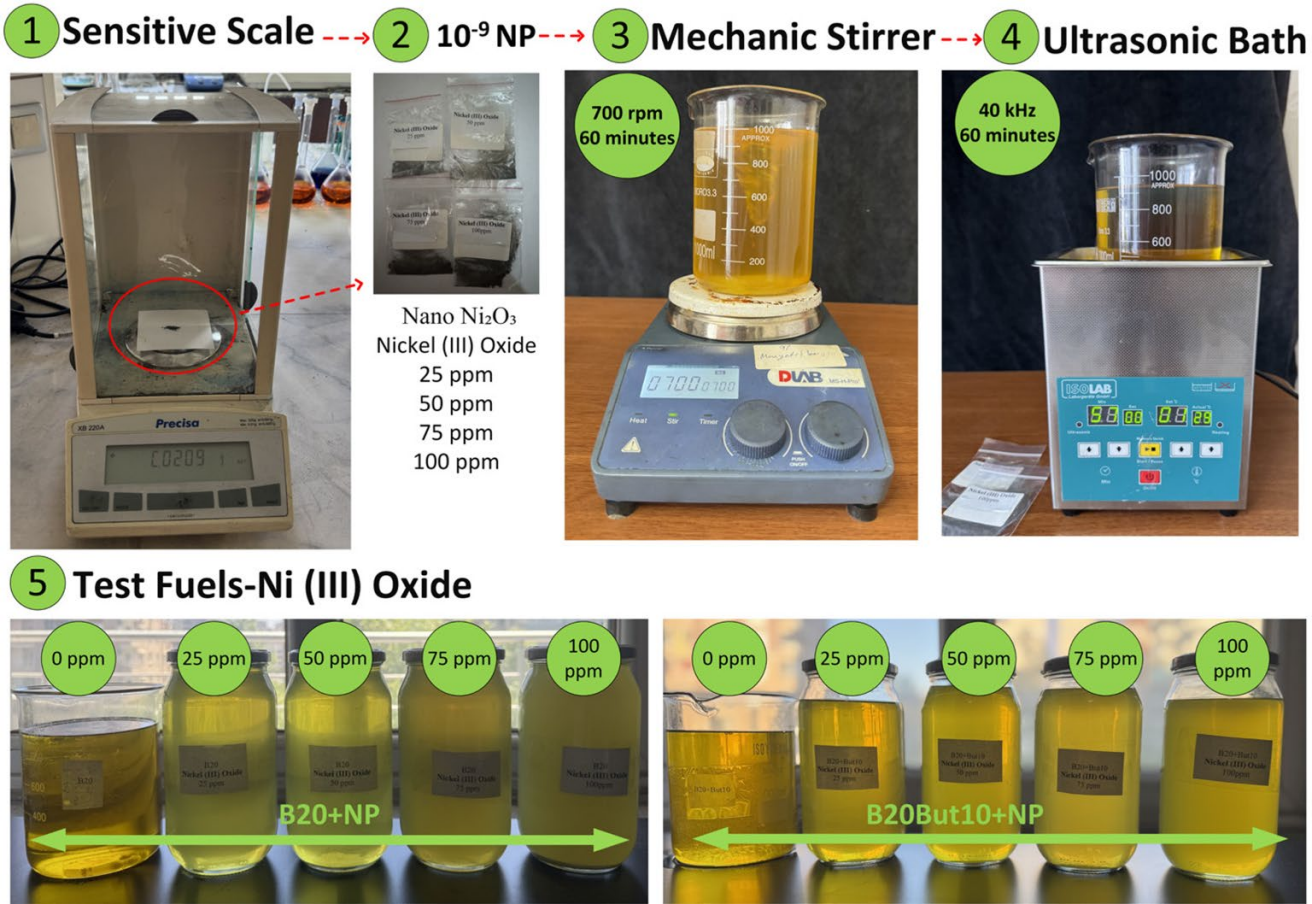
Schematic illustration of the preparation process.

For the preparation of the test fuels, a volumetric blending methodology compatible with conventional liquid fuel preparation processes was employed. Within this framework, biodiesel–n-butanol blends containing Ni₂O₃ were processed through a multi-stage dispersion technique. The specified NP quantities were first measured using a Precisa XB220A analytical balance and introduced into the fuel. The mixtures were then subjected to mechanical agitation in a DLAB MS-H-PRO magnetic stirrer at 700 rpm for 60 min to achieve an initial dispersion. In the subsequent step, an ISOLAB ultrasonic bath operating at 40 kHz for 1 h was employed to further enhance particle distribution and improve suspension uniformity. As shown in Fig. 2, the sedimentation assessment revealed that both B20 + Ni₂O₃ and B20But10 + Ni₂O₃ blends maintained a visually homogeneous appearance during the early storage periods. No discernible sedimentation or phase separation was detected after 3 h and 3 days of quiescent storage, indicating sufficient short-term and mid-term dispersion stability relevant to engine testing conditions. However, after 6 weeks of storage, partial sedimentation became observable, suggesting that while the NP-enhanced blends exhibit adequate operational stability, long-term storage may promote gradual particle settling. It should be noted that minor color differences between the samples are solely attributed to lighting variations during image acquisition and do not reflect changes in fuel composition or dispersion quality. Experimental observations revealed that when the NP dosage exceeded 100 ppm, notable agglomeration occurred, leading to deterioration in the stability of the blends. Instability arises because higher particle densities strengthen van der Waals forces, promoting clustering and reducing suspension homogeneity. Such conditions hinder the atomization of fuel during injection, reduce combustion efficiency, and may even induce engine knocking^70^. Therefore, the upper concentration limit for NPs was restricted to 100 ppm. Consistent with reports in previous studies, maintaining NP addition within the 0–100 ppm range is considered essential for preserving engine reliability, avoiding injector blockage, and ensuring effective catalytic contribution while achieving stable performance^31,33,71^. Following preparation, the samples were transferred to transparent glass containers for visual inspection. Only fuels exhibiting no sedimentation or signs of aggregation were selected for testing. This protocol ensured that NP dispersion remained stable and that combustion, emission, and performance analyses were performed under standardized conditions. A schematic representation of the preparation procedure is provided in (Fig. 3).

The prepared fuel blends underwent physical and chemical characterization at the TÜBİTAK MAM laboratories in Istanbul, Turkey. The corresponding results obtained from the analyses of the five blends are provided in Table 3. With the addition of Ni₂O₃, significant changes occurred in the physical and chemical properties of both fuel types, and these changes were evaluated in accordance with ASTM standards. The analysis included key parameters such as density (kg/m^3^), lower heating value (MJ/kg), kinematic viscosity (cSt), flash point (°C), and cetane index, and the obtained results are presented in detail in Table 3.

**Table 3:** Properties of the biodiesel, n-butanol and NP fuel blends.

Properties	Method	B20	B20But10	B20	B20But10	B20	B20But10	B20	B20But10	B20	B20But10
		0 ppm		25 ppm		50 ppm		75 ppm		100 ppm	
Density, kg/m^3^	ASTM D5865	835.1	833.7	835.5	834.2	835.9	834.7	836.3	835.3	836.6	835.7
Calorific value, Mj/kg	ASTM D5865	41.24	39.41	40.29	38.42	39.81	38.15	39.37	37.56	38.82	37.18
Kinematic Viscosity, cSt	ASTM D5865	3.12	3.17	3.13	3.19	3.14	3.2	3.19	3.29	3.28	3.41
Flash Point, °C	ASTM D5865	68	44	67	44	66	43	66	43	65	42
Cetane Index	ASTM D613	52.1	46.8	52.4	47.1	53.1	47.9	53.8	48.7	54.1	49.3

An examination of the physicochemical properties of the test fuels presented in Table 3 shows that Ni₂O₃ exhibits similar effects on both fuel blends. As NP concentration increased, small but consistent rises in density and kinematic viscosity were recorded, which improved fuel atomization quality and droplet homogeneity after injection, in agreement with trends reported in the literature^35^. A decrease in flash point values was also observed, enabling the fuel to evaporate at lower temperatures and ignite more easily inside the cylinder. This finding is further supported by the increases in cetane index. The improvement in cetane index can be attributed to the high thermal conductivity and catalytic properties of Ni₂O₃, which reduce ignition delay and contribute to a more stable combustion process^39^. However, due to the non-combustible inorganic nature of the NPs, a noticeable reduction in calorific values was recorded for both fuel blends. Similar observations have also been reported in the literature, where metal oxide additives were found to lower the lower heating value of fuels^72^. When the fuel types are compared, the B20 blend exhibits a higher calorific value and cetane index than B20But10. This is attributed to the lower energy density of butanol. In addition, the low cetane number and low flame temperature of biobutanol lead to a longer ignition delay and lower in-cylinder pressure^73^. However, the B20But10 blend, with its lower flash point and higher volatility, facilitates ignitability, which can provide an advantage by accelerating the start of combustion under certain conditions.

### Experimental setup and methodology

The experimental program was carried out in the Engine Laboratory of Batman University, where prepared fuel blends were tested under controlled conditions. Throughout the study, the engine speed was kept constant at 1500 rpm, while the applied loads were gradually adjusted using a water-cooled eddy-current dynamometer (Baturalp Taylan type). This system, operated through ICEngineSoft engine performance analysis software (Version 9.0)^74^, not only allowed smooth regulation of engine load but also enabled continuous monitoring and recording of performance and emission parameters.

A single-cylinder, four-stroke diesel engine with direct injection and adjustable compression ratio (Kirloskar Oil Engines Ltd., India) was selected as the test platform. Detailed specifications of the engine are summarized in Table 4. Airflow into the engine was quantified using an orifice meter–manometer assembly attached to a calibrated air tank, ensuring accurate control of the air–fuel mixture. Simultaneously, intake and exhaust gas temperatures were tracked with Radix K-type thermocouples, supplying thermal data essential for interpreting combustion efficiency.

**Table 4:** Engine specification.

Description	Specification
Engine supplier	Apex Innovations Pvt. Ltd
Brand-Model	Kirloskar-TV1
Ignition	Compression-ignition
Injection	Direct-injection
Number of cylinder	1
Number of strokes per cycle	4
Bore (mm)-Stroke (mm)	87.5–110
Swept volume (cc)	661.45
Compression ratio	17.5:1
Maximum power (kW)-Speed (rpm)	5.2 @1500
Dynamometer	Eddy current type Brand: SAJ test plant pvt. Ltd
Connecting rod length (mm)	185
Cooling system	Water cooled
Intake system	Naturally aspirated
Fuel injection timing (o CA)	0–23° CA before bTDC (nominal)
Nozzle opening pressure (bar)	210
Injector nozzle number	4
Injection hole diameter	0.25 mm
Fuel type	Diesel
Fuel tank capacity (L)	15
Pressure transducer	Piezoelectric type, Brand: Kistle
Crank angle sensor	Optical encoder type, Brand: Kübler
Fuel injector pressure sensor	Piezoelectric ICP type, Brand: PCB Piezotronics Inc
Temperature Sensor	Thermocouple type K and RTD PT100, Brand: Radix; K-type range

**Fig. 4:**
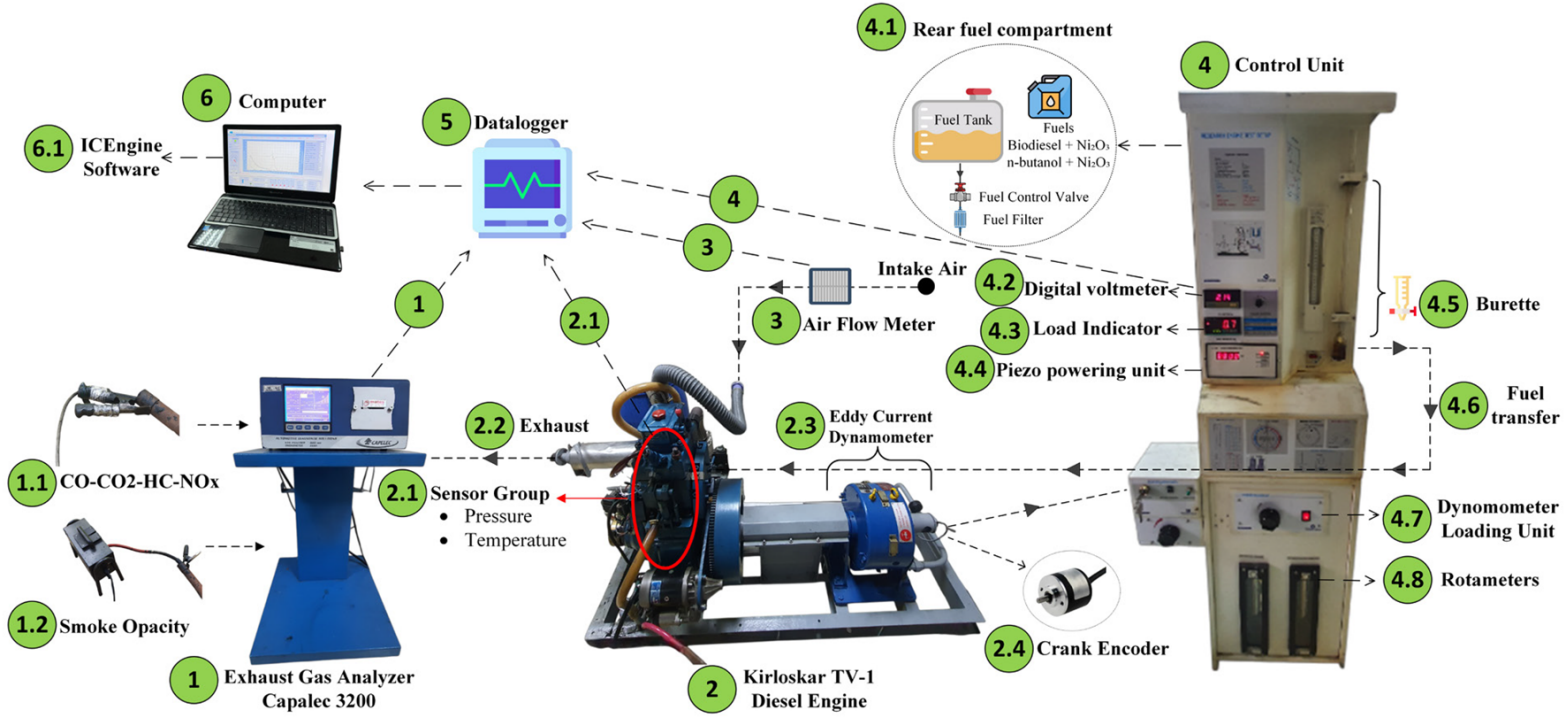
Experimental engine test setup schematic.

Fuel consumption was measured separately by recording the time required for the engine to use 100 mL of fuel from a volumetric burette system. This procedure, repeated at different operating points, provided a precise and reproducible basis for evaluating brake-specific fuel consumption. In addition, in-cylinder pressure traces and crank-angle information were obtained with a piezoelectric pressure sensor and an optical encoder, interfaced with a National Instruments NI USB-6210 data acquisition unit. These measurements formed the basis for determining ignition delay and heat release behavior. Emission analysis was performed with a CAPALEC CAP 3200 gas analyzer, which delivered quantitative data on NOx, HC, CO, smoke and CO_2_. Exhaust gases were released via a stainless-steel pipe, and their temperature was continuously recorded to validate emission measurements. Reference runs were conducted first with biodiesel and n-butanol blends, followed by experiments using NP-enriched fuels, thereby ensuring a systematic comparison of baseline and modified fuels under identical conditions. The schematic representation of the test rig and its principal elements is illustrated in (Fig. 4).

In the present study, the test procedure was carried out under steady engine speed while varying the applied load conditions. Calibration and validation of all sensors, filters, and measuring devices were carried out in compliance with international guidelines before each experiment. To maintain measurement consistency, the engine was allowed to return to ambient temperature whenever a new fuel blend was introduced; at the same stage, the fuel filter was cleaned and the filters of the exhaust gas analyzer were replaced. The load on the engine was controlled through a precision eddy-current dynamometer, which enabled stable operation and a systematic evaluation of engine performance under different load settings. Cylinder pressure and crank-angle signals were collected and averaged over ten successive cycles for every test point, and these datasets were processed using ICEngineSoft v9.0 software to extract detailed combustion characteristics. Each test sequence was repeated three times, and the mean values were taken into account in order to minimize experimental uncertainty and to increase the reliability of the results.

## Calculation method

The HRR is determined by applying the first law of thermodynamics to the in-cylinder gases during the combustion process^31^. According to the first law of thermodynamics;

1 $$\frac{dU}{dt}=\dot{Q}-\dot{W}$$

2 $$m{C}_{v}\frac{dT}{dt}=\dot{Q}-P\frac{dV}{dt}$$

Here, *Q* represents the net rate of heat addition, encompassing both the heat released during combustion and the heat transferred through the cylinder walls, while *W* denotes the rate of work performed by the system as a result of boundary volume changes. For simplification of Eq. (2), the working fluid can be assumed to behave as an ideal gas.

3 $$PV=mRT$$

Assuming a constant mass within the cylinder, Eq. (3) is written as^31^

4 $$\frac{dT}{dt}=\frac{1}{mR}\left[P\frac{dV}{dt}+V\frac{dP}{dt}\right]$$

When Eq. (2) and Eq. (3) are combined, the heat release equation is as follows:

5 $$\dot{Q}=\left[\frac{{C}_{v}}{R}+1\right]P\frac{dV}{dt}+\frac{{C}_{v}}{R}V\frac{dP}{dt}$$

When time (t) is replaced by crank angle (h);

6 $${Q}_{t}=\frac{k}{k-1}P\frac{\partial V}{\partial \theta }+\frac{1}{1-k}V\frac{\partial P}{\partial \theta }$$

Here, P denotes the CP, V is the cylinder volume, h represents the crank angle, and k is the specific heat ratio, taken as 1.35 for the diesel heat release analysis.

The CHRR was calculated for the tested fuels as the integral of the heat release rate over the defined crank angle interval;

7 $${Q}_{cum}=\int P\frac{k}{k-1}\frac{\partial V}{\partial \theta }+\int V\frac{1}{1-k}\frac{\partial P}{\partial \theta }$$

The start of combustion (SOC) is identified as the crank angle at which the HRR curve passes from negative to positive values^19^. The start of injection (SOI) is defined as the crank angle position where the fuel injector begins to operate. The ID is then determined as the time interval, expressed in crank angle degrees, between SOI and SOC.

Engine power is defined as the effective force transmitted to the crankshaft, representing the useful mechanical output of the engine. Based on this definition, the key engine performance parameters are formulated as follows^75,76^;

8 $${P}_{b}=\frac{2\pi .\omega .\tau }{1000}$$

Here, $\tau$ is the brake torque (Nm), $\omega$ is the crankshaft rotation speed (rpm), and ${P}_{b}$ (kW) is the brake power. To evaluate engine performance, BMEP, BSFC, and BTE were calculated. Equation (2) and (3) were used to derive these expressions^31,76,77^;

9 $$BMEP (Pa)=\frac{{P}_{b}{.n}_{r}}{{V}_{d}.\omega }{10}^{3}$$

10 $$BSFC \left(\frac{g}{kW}.h\right)=\frac{{\dot{m}}_{f}}{{P}_{b}}{10}^{3}$$

11 $$BTE=\frac{{P}_{b}}{{\dot{m}}_{f}.{Q}_{LHV}}3600$$

Here, ${n}_{r}$ is the crank speed for one complete cycle,${V}_{d}$ is the displacement volume of the cylinders. ${\dot{m}}_{f} (\frac{kg}{h})$ fuel mass flow rate, ${Q}_{LHV}(kJ/kg)$ is the lower heating value of the fuel.

### Uncertainty assessment

To quantify the reliability of the experimental results, an uncertainty analysis was conducted using the methodology proposed by Holman^78^. In this approach, the target variable *R* is influenced by a set of *n* independent variables, denoted as *x*, and the combined uncertainty is evaluated through error propagation techniques. Equation (12) provides the mathematical formulation for determining the uncertainty associated with each contributing parameter. The accuracy and calibration precision of the instruments, along with environmental conditions, are key contributors to overall uncertainty. This analysis not only quantifies the confidence level of the experimental data but also highlights the variables with the greatest impact on measurement reliability. Each measurement parameter’s corresponding uncertainty value is presented in detail in (Table 5).

12 $${U}_{R}={\left[{\left(\frac{{x}_{1}}{R}\frac{\partial R}{\partial {x}_{1}}{U}_{1}\right)}^{2}+{\left(\frac{{x}_{2}}{R}\frac{\partial R}{\partial {x}_{2}}{U}_{2}\right)}^{2}+\dots +{\left(\frac{{x}_{n}}{R}\frac{\partial R}{\partial {x}_{n}}{U}_{n}\right)}^{2}\right]}^\frac{1}{2}$$

13 $$\begin{aligned} \Delta \user2{U}_{{{\mathbf{Overall}}}} & = \sqrt {\left( {\Delta \user2{U}_{{\user2{BP}}} } \right)^{2} + \left( {\Delta \user2{U}_{{\user2{MFC}}} } \right)^{2} + \left( {\Delta \user2{U}_{{\user2{BTE}}} } \right)^{2} + \left( {\Delta \user2{U}_{{\user2{CO}}} } \right)^{2} + \left( {\Delta \user2{U}_{{\user2{HC}}} } \right)^{2} + \left( {\Delta \user2{U}_{{\user2{NOx}}} } \right)^{2} + \left( {\Delta \user2{U}_{{\user2{Smoke}}} } \right)^{2} + \left( {\Delta \user2{U}_{{\user2{CO}_{2} }} } \right)^{2} } \\ & = \sqrt {\left( {0.84} \right)^{2} + \left( {1.55} \right)^{2} + \left( {1.76} \right)^{2} + \left( {0.84} \right)^{2} + \left( {0.83} \right)^{2} + \left( {0.83} \right)^{2} + \left( {0.83} \right)^{2} + \left( {0.84} \right)^{2} } \\ & = 3.11\user2{~\% } \\ \end{aligned}$$

**Table 5:** Accuracy specifications and uncertainty levels of measurement systems and calculated outputs.

Parameter	Measurement range	Accuracy	Uncertainty (%)
Brake power	0–3.5 kW	± 0.3 kW	± 0.84
Engine speed	0–1500 rpm	± 0.1 rpm	± 0.07
Engine load	0–12 kg	± 0.1 kg	± 0.83
Mass of the fuel consumption	-	-	± 1.55
Burette system	-	± 0.1 cc	± 1.0
Digital watch	-	± 0.2 s	± 0.3
Brake thermal efficiency	-	-	± 1.76
Brake specific fuel consumption	-	-	± 1.76
CO	0–10%	± 0.01%	± 0.84
HC	0–20.000 ppm	± 1.0 ppm	± 0.83
NO_x_	0–5000 ppm	± 1.0 ppm	± 0.83
Smoke	0–99.9% vol	± 0.01%	± 0.83
CO_2_	0–10%	± 0.01%	± 0.84

Based on the calculation method defined in Eq. (13), the combined uncertainty for the parameters characterizing system performance and emissions was determined to be ± 3.11%. It should be emphasized that several performance and combustion indicators reported in this study are not directly measured quantities but are derived from multiple primary measurements. Consequently, their associated uncertainties inherently reflect the cumulative propagation of uncertainties originating from several measurement sources rather than isolated instrumental errors.

### Response surface methodology

RSM is a statistical optimization tool used to explore the relationships between multiple input variables and one or more output responses. The methodology generally consists of experimental planning, model fitting, statistical validation, and optimization. In the present study, NP concentration, BMEP, and emission values were defined as independent variables, while brake thermal efficiency BTE and BSFC were selected as the responses. A two-factor asymmetric full factorial design was applied to accommodate different levels of input variables, generating 20 experimental runs in a 4 × 5 matrix format. Second-order regression models were constructed for each response using Eq. (14), allowing for a detailed assessment of variable effects and system optimization^77,79,80^.

14 $$Y={\beta }_{0}+{\sum }_{i=1}^{n}{\beta }_{i}{x}_{i}+{\sum }_{i=1}^{n}{\beta }_{ii}{{x}_{i}}^{2}+{\sum }_{i>j}^{n}{\beta }_{ij}{x}_{i}{x}_{j}+\varepsilon$$

In this model, *Y* denotes the predicted response value, n represents the total number of independent variables, and ${\beta }_{0}$ is the intercept term. The coefficients ${\beta }_{i}$, ${\beta }_{ii}$ and ${\beta }_{ij}$ correspond to the linear, quadratic, and interaction effects of the factors ${x}_{i}$ and ${x}_{j}$ respectively. The term $\varepsilon$ accounts for the random error associated with the response.

In this optimization study, two primary input factors were considered: NP concentration and engine load. Ni₂O₃ concentration was varied at five levels (0, 25, 50, 75, and 100 ppm) to represent low, medium, and high additive dosages. Similarly, engine load, expressed in terms of BMEP, was examined at 0.3, 1.0, 2.0, 3.0, and full load to capture operating conditions from low to high severity. The selected ranges enabled a systematic assessment of the fuels’ combustion, performance, and emission behavior under diverse conditions.

A central composite design (CCD) framework was employed within the MINITAB environment to generate the experimental matrix and model potential factor interactions. In total, 20 operating conditions were established, as detailed in Table 6. The RSM approach was then applied to evaluate the combined influence of NP concentration and BMEP on key engine responses, including BTE, BSFC, CO, HC, NOₓ, smoke opacity and CO₂ emissions. To ensure robustness, the experimental dataset was analyzed through Analysis of Variance (ANOVA), which provided statistical verification of the model’s predictive capability and its effectiveness in capturing both linear and non-linear interactions between input factors.

**Table 6:** RSM design, factors and levels.

Method	Central composite full factorial design
Continuous factors	NP amount & BMEP
Terms & confidence levels	Full quadratic & 95%
NP amount (ppm) levels	0; 25; 50; 75; 100
BMEP (bar) levels	0.3; 1.0; 2.0; 3.0

In Table 7, five separate optimization models were formulated to capture different operational goals and priorities. Each model was designed by assigning varying levels of importance to the response variables, thereby representing distinct optimization strategies. Model 1 follows a balanced scheme, giving equal weight to all responses. Model 2 is oriented toward environmental considerations, emphasizing only emission related parameters. Model 3 focuses exclusively on engine performance metrics, disregarding emission related factors, with operational efficiency and power output defined as the primary objectives. Model 4 integrates all response variables but places greater emphasis on performance indicators, whereas Model 5 also accounts for all responses comprehensively while prioritizing emission criteria. These modeling approaches enabled the determination of optimum NP dosage (ppm) and engine load (BMEP, bar) values under different operating conditions and design requirements. Consequently, flexible and goal oriented optimization pathways were proposed for both short and long term applications. In all cases, the overarching objective was to maximize engine performance while reducing harmful exhaust emissions.

**Table 7:** Optimization criteria for each model.

Inputs		NP {0, 25,50,75,100} – BMEP {0.3, 1.0, 2.0, 3.0}									
Constant	Target	Model 1		Model 2		Model 3		Model 4		Model 5	
BTE (%)	Max	✓	*	-	-	✓	*	✓	***	✓	*
BSFC (kg/kWh)	Min	✓	*	-	-	✓	*	✓	***	✓	*
CO (%)	Min	✓	*	✓	*	-	-	✓	*	✓	***
NOx (ppm)	Min	✓	*	✓	*	-	-	✓	*	✓	***
HC (ppm)	Min	✓	*	✓	*	-	-	✓	*	✓	***
Smoke (m-1)	Min	✓	*	✓	*	-	-	✓	*	✓	***
CO_2_ (%)	Min	✓	*	✓	*	-	-	✓	*	✓	***

## Result and discussion

The exhaust emissions, combustion and engine performance of B20 and B20But10 fuel blends prepared with Ni₂O₃ at different concentrations (25, 50, 75, and 100 ppm) were systematically investigated under varying engine loads (0.3, 1.0, 2.0, and 3.0 bar BMEP). The experimental data obtained were compared with the neat B20 and B20But10 blends without additives in order to more comprehensively evaluate the effects of NP addition on engine behavior.

### Combustion performance

The combustion characteristics of the tested fuels were evaluated at different engine loads to better understand the thermodynamic and chemical processes taking place in the cylinder. Key parameters derived from in-cylinder measurements included the start of injection (SOI, °CA)**,** start of combustion (SOC, °CA)**,** ignition delay (ID, °CA), crank angle of peak pressure (ACP_max_, °CA)**,** maximum cylinder pressure (CP_max_, bar)**,** crank angle of maximum apparent heat release rate (AHRR_max_, °CA), peak apparent heat release rate (HRR_max_, J/°CA)**,** cumulative heat release (CHRR_max_, J)**,** crank angle of maximum cumulative heat release rate (ACHRR_max_, °CA)**,** peak pressure rise rate (PRR_max_, bar/°CA)**,** and angle of maximum instantaneous power (AIP_max_, °CA)**.** These parameters were systematically analyzed and compared with relevant findings from the literature to interpret the influence of NP enriched fuel blends on engine combustion behavior. The numerical results corresponding to these parameters are presented in Table 8.

**Table 8:** Combustion performance of test fuels at different engine loads and dosages.

BMEP (bar)	Fuel types	SOI (deg)	SOC (deg)	ID (deg)	ACP_max_ (deg)	CP_max_ (bar)	AHRR_max_ (deg)	HRR_max_ (J/deg)	ACHRR_max_ (deg)	CHRR_max_ (J)	AIP_max_ (deg)	PRR_max_ (bar/deg)
0.3	B20	334	348	14	367	43.78	359	14.79	496	932	359	1.82
	B20 + 25 ppm	334	348	14	368	44.37	359	15.21	493	929	359	1.85
	B20 + 50 ppm	334	347	13	368	45.19	360	15.97	494	930	359	1.91
	B20 + 75 ppm	334	347	13	367	46.03	359	16.54	492	929	359	1.95
	B20 + 100 ppm	334	347	13	367	46.62	360	16.95	492	930	360	1.98
	B20But10	334	349	15	367	43.51	358	14.82	497	931	358	1.7
	B20But10 + 25 ppm	334	348	14	368	44.13	359	15.33	495	929	358	1.74
	B20But10 + 50 ppm	334	347	13	367	45.07	358	16.07	495	930	359	1.72
	B20But10 + 75 ppm	334	348	14	367	45.95	358	16.51	494	930	358	1.81
	B20But10 + 100 ppm	334	348	14	367	46.23	358	17.24	495	932	358	1.85
1	B20	334	347	13	366	47.49	358	18.14	490	945	358	2.02
	B20 + 25 ppm	334	347	13	367	47.63	359	18.55	487	942	358	2.05
	B20 + 50 ppm	335	347	12	367	48.52	360	19.01	487	944	359	2.13
	B20 + 75 ppm	334	346	12	366	49.75	358	19.54	488	945	358	2.18
	B20 + 100 ppm	334	346	12	366	49.88	358	20.43	487	944	358	2.23
	B20But10	334	348	14	365	46.88	358	18.57	491	949	357	2.02
	B20But10 + 25 ppm	334	347	13	366	47.21	357	18.92	490	948	357	2.09
	B20But10 + 50 ppm	334	348	14	365	47.15	357	19.35	491	949	357	2.14
	B20But10 + 75 ppm	335	348	13	365	48.68	358	20.18	492	951	357	2.13
	B20But10 + 100 ppm	334	347	13	366	49.23	357	21.09	491	951	358	2.17
2	B20	335	348	13	366	49.98	357	22.19	486	959	358	2.47
	B20 + 25 ppm	335	347	12	365	50.47	358	22.86	483	956	358	2.52
	B20 + 50 ppm	334	346	12	365	51.09	357	23.42	484	959	357	2.55
	B20 + 75 ppm	335	346	11	365	51.78	357	23.97	484	960	358	2.7
	B20 + 100 ppm	335	346	11	365	52.67	358	24.81	483	959	358	2.79
	B20But10	335	348	13	365	49.44	357	22.18	488	965	357	2.46
	B20But10 + 25 ppm	335	348	13	366	50.12	357	23.01	486	964	356	2.49
	B20But10 + 50 ppm	334	347	13	366	50.95	357	23.92	486	964	357	2.54
	B20But10 + 75 ppm	335	347	12	365	51.63	356	24.78	487	967	357	2.57
	B20But10 + 100 ppm	335	346	11	366	52.51	356	25.13	487	968	356	2.61
3	B20	336	347	11	365	53.03	357	26.54	483	979	357	3.22
	B20 + 25 ppm	336	346	10	365	53.77	357	27.41	481	977	357	3.24
	B20 + 50 ppm	335	346	11	365	54.26	357	27.96	479	975	356	3.25
	B20 + 75 ppm	336	346	10	365	54.81	357	28.38	480	978	357	3.31
	B20 + 100 ppm	336	346	10	364	55.86	357	29.45	479	976	356	3.34
	B20But10	335	347	12	366	52.21	354	27.7	484	981	354	3.05
	B20But10 + 25 ppm	335	347	12	366	53.07	354	28.11	483	980	354	3.11
	B20But10 + 50 ppm	335	346	11	366	53.5	355	28.8	484	982	355	3.14
	B20But10 + 75 ppm	335	346	11	365	54.27	355	29.37	482	978	355	3.19
	B20But10 + 100 ppm	335	345	10	365	55.45	355	30.02	483	981	355	3.25

In the evaluation of the combustion process, SOI, SOC and ID are considered critical parameters. ID refers to the period between SOI and SOC and directly indicates the ignition quality of the engine. Experimental findings showed that the ID, which was 14–15°CA for the neat fuels, decreased to approximately 10°CA at full load with the addition of 100 ppm NPs for both fuels. Moreover, across all loads, B20 consistently exhibited shorter ID values compared to B20But10. For example, under low-load conditions, the ID of B20 was 1–2°CA shorter, while at 2.0 bar BMEP the difference remained around 1°CA. At 3.0 BMEP, both fuels reached minimum values, although B20 still presented shorter delay periods. This trend can be explained by the higher cetane number and lower latent heat of vaporization of B20, which accelerate the preparation phase of combustion. By contrast, ternary blends, particularly with n-butanol addition, are known to extend ID periods due to their lower cetane number and higher latent heat of vaporization^81,82^. The addition of Ni₂O₃ contributed to the shortening of ID for both fuels, especially under high load conditions, thereby narrowing the gap between B20 and B20But10. Indeed, previous studies have also reported that NP additives shorten evaporation time, reduce ID, improve combustion efficiency, and lower fuel consumption^83,84^. Furthermore, as engine load increases, in-cylinder temperature and pressure rise, further reducing ID periods, which is consistent with findings in the literature^85^. The reduction in ID and the observed shifts in HRR behavior indicate a moderate advancement of the combustion process with increasing load and NP concentration. This advancement suggests that the combustion phasing, particularly the CA50 (defined as the crank angle at which 50% of the total heat is released), moved toward a more optimal position closer to top dead center (TDC) without becoming excessively advanced. The fact that peak HRR and maximum CP occurred shortly after TDC confirms that the main combustion event remained within a favorable crank angle window for efficient energy conversion and enhanced thermal efficiency. As shown in Table 8, injection timing was not independently optimized for each fuel blend, and the start of injection occurred at approximately 23–26° BTDC with minor shifts as BMEP increased, reflecting the inherent operating characteristics of the engine under different load conditions. Therefore, the observed combustion phasing reflects the combined influence of fuel properties and the existing injection strategy, rather than fuel effects alone.

### In-cylinder gas pressure

**Fig. 5:**
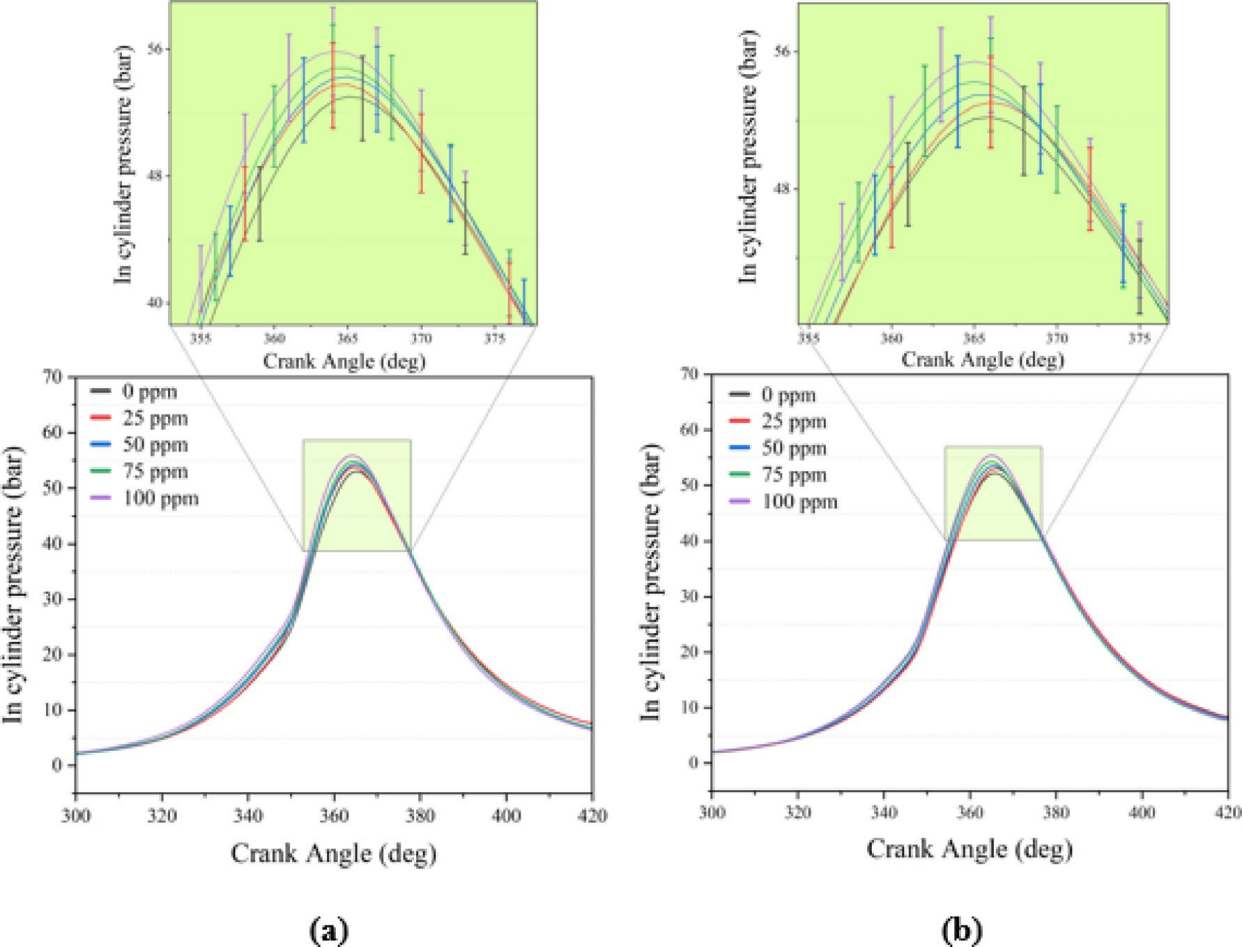
The in cylinder pressure curves at 3.0 BMEP loads (**a**) B20 + NP (**b**) B20But10 + NP.

CP analysis provides critical insights into the combustion phasing and heat release behavior of the tested fuels. As shown in Fig. 5 and summarized in Table 8, the CPₘₐₓ values of both B20 and B20But10 increased consistently with engine load; however, B20 produced higher peak pressures than B20But10 under all load conditions. At 0.3 BMEP, the neat B20 reached approximately 43.78 bar, whereas B20But10 was about 43.51 bar. Previous studies have reported lower maximum pressures for diesel/biodiesel blends containing n-butanol compared to neat diesel^14,86^. This has been attributed to the lower cetane number and higher latent heat of vaporization of butanol, which extend ID and shift the combustion phasing toward later crank angles^87^. Conversely, some research indicates that n-butanol addition can increase in-cylinder peak pressures when used with biodiesel, as the higher oxygen content and stronger premixed combustion phase promote higher CP values^88^. This effect is linked to the additional oxygen provided by n-butanol, which enhances mixture homogeneity and accelerates energy release during ignition. Moreover, the influence of n-butanol on CP has been shown to vary with fuel composition and combustion conditions^89^.

The addition of Ni₂O₃ notably improved the pressure characteristics of B20But10, narrowing the difference between the two fuels across all loads. The most pronounced effect was observed at full load, where with 100 ppm NP addition CP_max_ reached 55.86 bar for B20 and 55.45 bar for B20But10. These findings confirm the potential of Ni₂O₃ to enhance the combustion characteristics and overall engine performance of B20 and B20But10 blends. Researchers have reported that adding metal oxide NPs promotes greater fuel accumulation during the premixed combustion stage, leading to higher in-cylinder pressures and faster heat release rates^20^. Overall, the results show that while B20 inherently benefits from its higher cetane index and calorific value, B20But10 can largely compensate for the disadvantages introduced by n-butanol through the catalytic effect of Ni₂O₃, which shortens ID and accelerates the premixed combustion phase.

### Apparent heat release rate

**Fig. 6:**
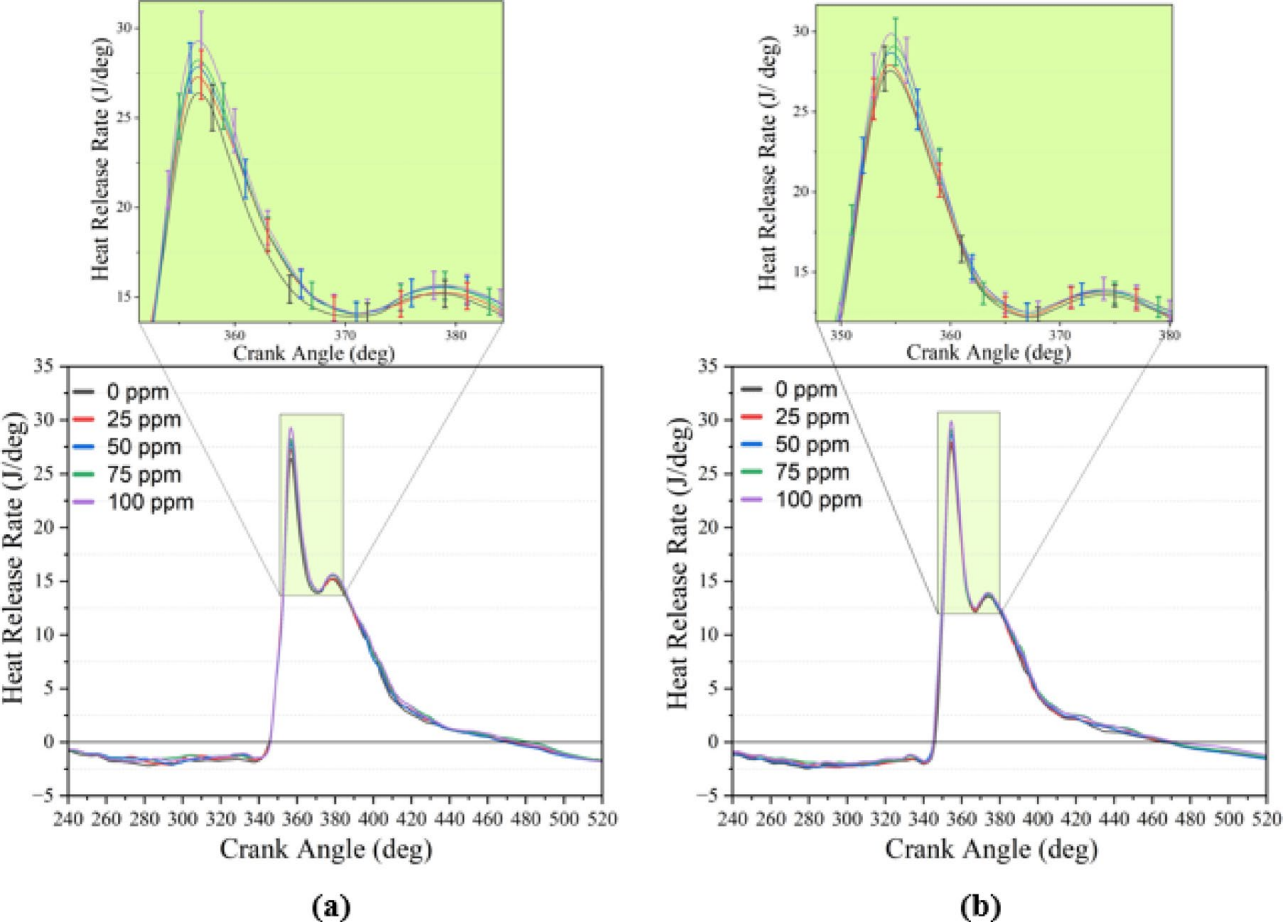
Heat release rate curves at 3.0 BMEP loads (**a**) B20 + NP (**b**) B20But10 + NP.

HRR provides a quantitative assessment of the total energy released over the crank angle and enables tracking of the premixed, rapid, controlled, and late combustion phases. All experiments were conducted with a fixed injection timing of 23° before top dead center (BTDC). Injection timing was not independently optimized for each fuel blend; therefore, the HRR results reflect comparative combustion behavior under a uniform injection strategy rather than fuel-specific optimized conditions. The HRR results obtained for different loads and NP dosages are summarized in Table 8, and the curves at full load are presented in (Fig. 6). The study revealed that HRR increased steadily with load, and B20But10 blends produced higher HRR_max_ values than B20 under the same conditions. At full load, HRR_max_ increased from 26.54 to 29.45 J/°CA for B20 and from 27.70 to 30.02 J/°CA for B20But10. The locations of AHRR_max_ were generally clustered in the 354–360°CA range; notably, for B20But10, increasing load and NP concentration caused the peak points to occur at slightly earlier crank angles. This trend indicates that NP addition improves mixture homogeneity and strengthens energy release in the premixed phase, consistent with findings reported in the literature^90^.

According to the data in Table 3, B20 has a higher lower heating value and cetane index compared to B20But10; however, the oxygenated composition of B20But10 produces a steeper HRR slope and higher HRR_max_ during the premixed phase. This can be explained by the increased laminar flame speed provided by oxygen content and the lower boiling point facilitating earlier vaporization. Literature also reports that oxygenated components steepen HRR curves and accelerate early stage energy release at low and medium loads^91,92^. As engine load rises, in-cylinder temperature and pressure increase, promoting faster combustion and higher HRR; thus, the load-HRR growth observed in this study is consistent. The tendency of higher compression ratio and load to increase HRR has also been emphasized in other studies^20^. Additionally, the addition of n-butanol, due to its oxygenated structure and relatively low cetane number, increases fuel accumulation in the premixed phase, thereby elevating the HRR peak^93^. The further increase in accumulation and peak intensity observed here with NP addition is also consistent with previous findings^94^.

### Cumulative heat release rate

**Fig. 7:**
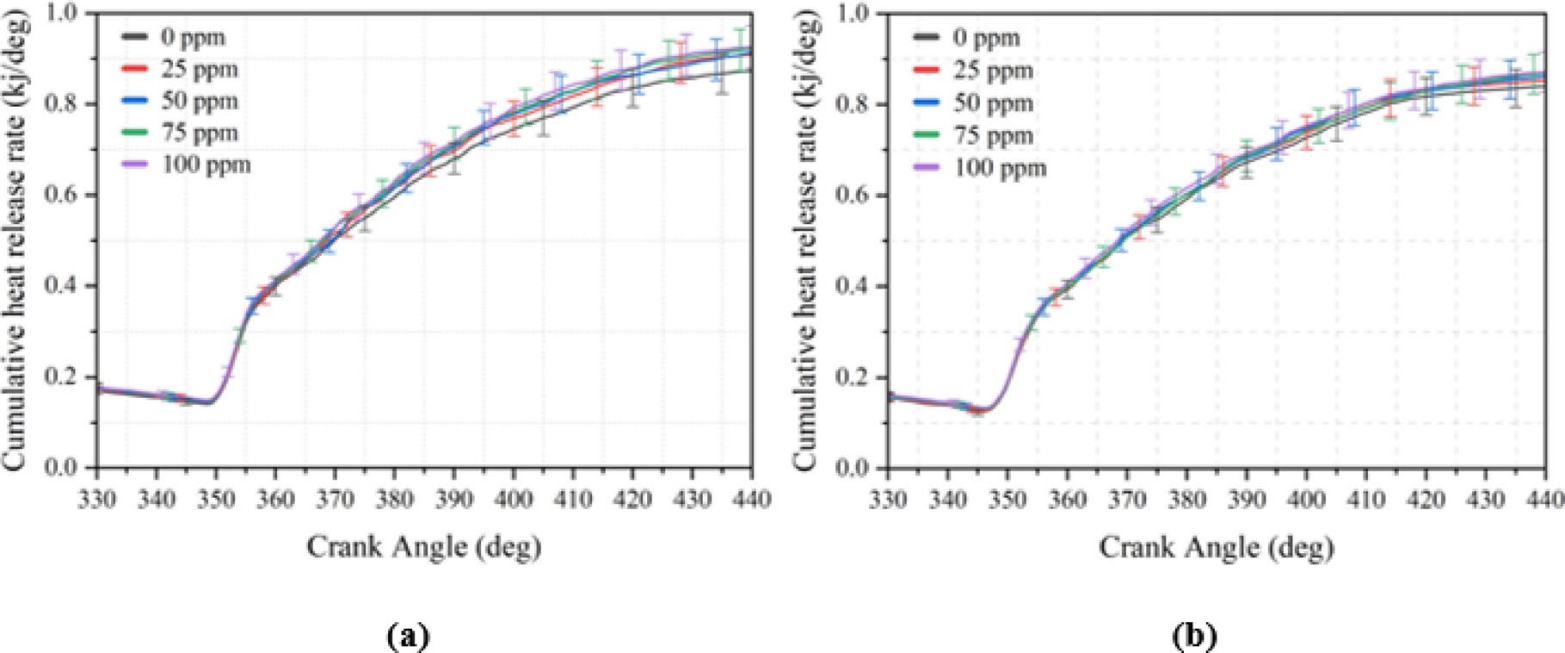
Cumulative heat release rate curves at 3.0 BMEP loads (**a**) B20 + NP (**b**) B20But10 + NP.

CHRR represents the total energy released during combustion as a function of crank angle, obtained by integrating the instantaneous heat release over the cycle. In this study, CHRR results for all engine loads and NP concentrations are comprehensively presented in Table 8, while Fig. 7 illustrates the full load condition for B20 and B20But10 blends. The findings reveal that B20 consistently exhibits higher CHRR values than B20But10, which can be attributed to its higher calorific value and cetane index, resulting in more efficient oxidation and greater cumulative energy release^95^. By contrast, B20But10 blends, particularly at early crank angles, show relatively lower CHRR due to the combined effects of butanol’s lower energy density, higher latent heat of vaporization, and intrinsic oxygen content, all of which prolong the evaporation phase and delay effective heat release^86^. This effect is further reinforced by the relatively higher viscosity and density of biodiesel based fuels, which reduce atomization efficiency and suppress cumulative energy release at low and medium loads.

However, under elevated load conditions, the oxygen enrichment of butanol becomes more beneficial, as the increased in-cylinder temperatures enhance the reactivity of oxygenated species. This accelerates oxidation reactions and contributes to more stable combustion, thereby partially compensating for the initial disadvantage^96^. Moreover, the addition of Ni₂O₃ lowers the activation energy for ignition and promotes catalytic pathways, leading to improved combustion completeness. The overall improvement in CHRR can therefore be explained by the combined effect of NP induced property enhancements and the oxygenated nature of butanol, which becomes advantageous at higher loads^97,98^.

### The rate of pressure rise

**Fig. 8:**
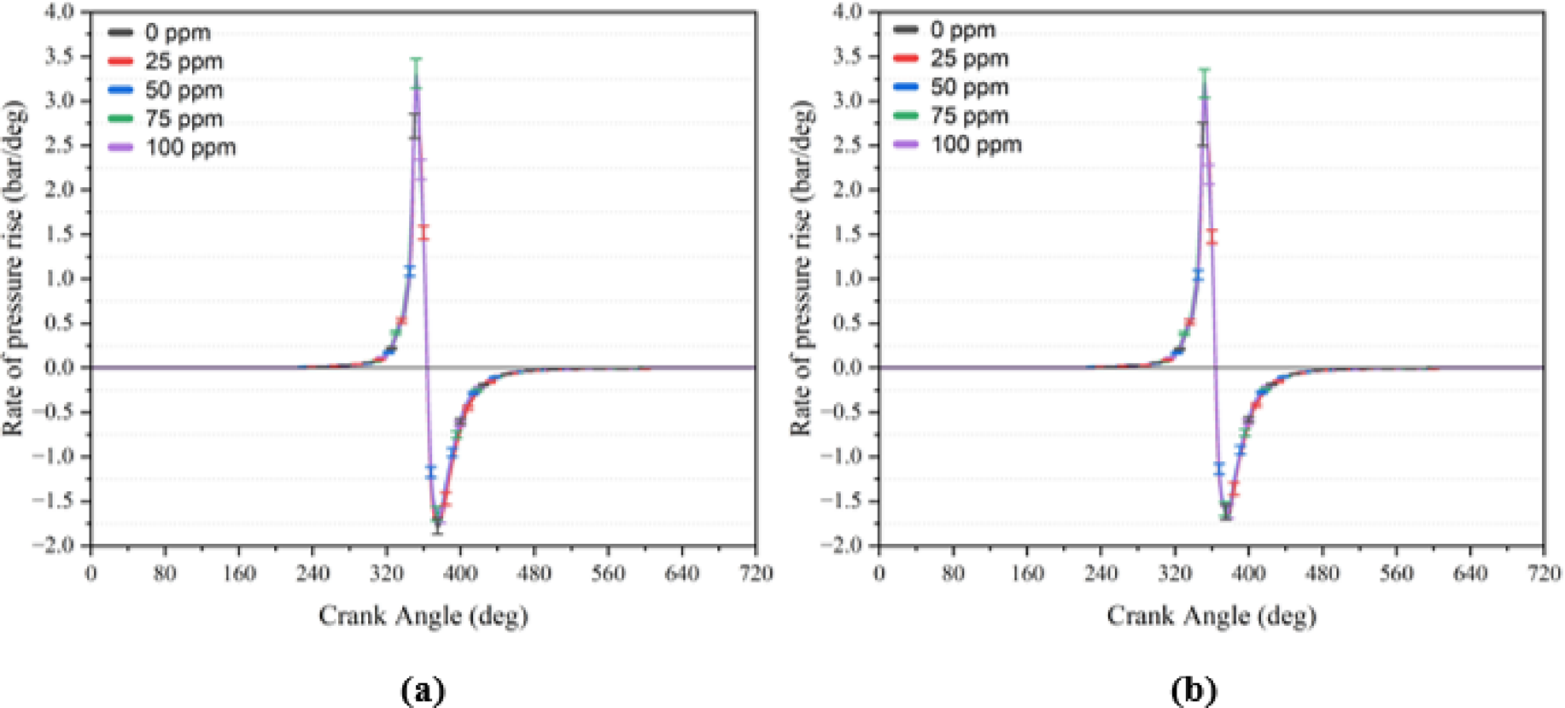
The rate of pressure rise curves at 3.0 BMEP loads (**a**) B20 + NP (**b**) B20But10 + NP.

PRR is defined as the change in in-cylinder pressure per crank angle degree and is a key indicator of combustion stability, vibration, and knock tendency^24^. PRR results for all engine loads and NP concentrations are presented in Table 8, while Fig. 8 shows the variation under full load conditions for B20 and B20But10 blends. The results demonstrate that engine load is the dominant factor: for example, PRR for B20 increased from 1.82 bar/°CA at 0.3 BMEP to 3.22 bar/°CA at 3.0 BMEP, consistent with reports that higher loads produce steeper pressure rise rates^99^.

Fuel composition produced only minor differences. At full load, the lowest PRR was recorded for B20But10 (3.05 bar/°CA), whereas B20 with 100 ppm additive reached the highest value (3.34 bar/°CA). The slightly reduced PRR of B20But10 can be linked to butanol’s lower cetane number and higher latent heat of vaporization, which extend ID and moderate the premixed combustion phase. Conversely, the increase in B20 with NPs suggests a faster combustion rate, aligning with studies showing that catalytic additives can accelerate combustion and raise PRR^100^. The pressure rise rate increased gradually with engine load and additive concentration; however, the relative change in pressure rise rate associated with fuel modification remained below approximately 5%. More importantly, the absolute pressure rise rate values observed under all test conditions were well within the ranges generally reported as safe for single-cylinder diesel engines. This indicates that the observed combustion acceleration did not induce knocking tendencies or excessive mechanical stress.

### Engine performance

Engine performance in this study has been evaluated mainly through BTE and BSFC. These two parameters serve as essential indicators for understanding both the energy conversion effectiveness and the fuel economy of the tested blends. The variations of BTE and BSFC under different engine loads and NP concentrations are presented in the following subsections, supported by experimental results and recent literature findings.

### Brake thermal efficiency

**Fig. 9:**
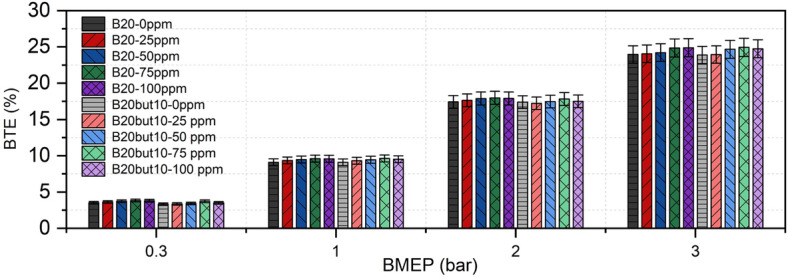
Variation of BTE with NP and BMEP.

BTE is a key indicator representing the conversion of the fuel’s chemical energy into mechanical power. As shown in Fig. 9, BTE increases with engine load for all fuels; this trend can be attributed to more effective combustion under higher in-cylinder temperature and pressure. In the neat fuel comparison, B20 slightly outperformed B20But10 across all loads; the addition of n-butanol limits efficiency due to its lower heating value and the cooling effect caused by its high latent heat of vaporization^14,40^. It has also been reported that diesel–biodiesel–alcohol ternary blends tend to show relatively lower BTE because of reduced calorific value and increased viscosity^82^. On the other hand, some studies have found that BTE can increase in butanol–diesel blends compared to neat diesel as the butanol fraction rises, which is attributed to the oxygen content of butanol facilitating more complete diffusion combustion, though its lower cetane number leads to longer ID and stronger premixed combustion^18^.

The interaction between NP dosage and engine load exhibited a clear threshold behavior. At 0.3 and 1.0 bar BMEP, BTE for both B20 and B20But10 increased gradually from 0 to 75 ppm, peaked at 75 ppm, and then showed a slight drop at 100 ppm. This small decline may be related to NP agglomeration and increased heat absorption at higher dosages, slightly weakening heat release and fluidity^76^. A similar “75 ppm optimum” was observed at 2.0 bar BMEP (B20: 17.98%; B20But10: 17.82%), while at 3.0 bar BMEP BTE reached the 24–25% range for both fuels, with B20But10 approaching B20 at 50–75 ppm. These results suggest that under high load, the combined effects of increased reactivity and improved mixture homogeneity with the catalytic and thermal conductivity contributions of NPs strengthen energy conversion, particularly in oxygenated blends^101^. Indeed, previous studies have reported that NPs shorten ID, accelerate oxidation, and promote more stable combustion; by encouraging micro explosions and vaporization, they reduce physical delay and enhance BTE^102^. Similar BTE improvements have also been reported with the addition of nickel oxide NPs, which improve atomization and heat/mass transfer due to their high surface area to volume ratio^42,54^.

### Brake specific fuel consumption

**Fig. 10:**
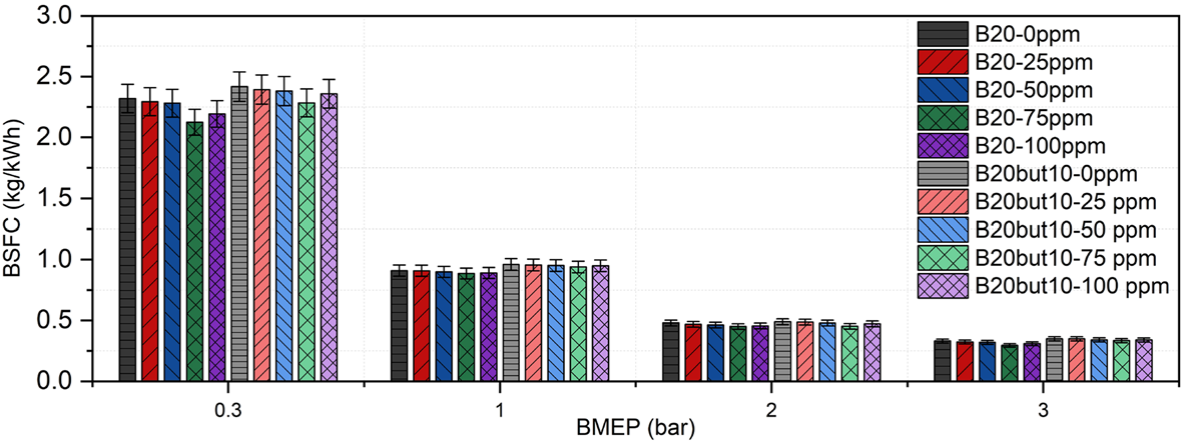
Variation of BSFC with NP and BMEP.

BSFC is a fundamental performance parameter representing the ratio of the mechanical power produced by an internal combustion engine to the energy content of the fuel consumed. In other words, BSFC is defined as the amount of fuel required to generate 1 kWh of power and directly reflects engine efficiency. Figure 10 presents the BSFC values of B20 and B20But10 fuel blends obtained under different load conditions. Experimental findings revealed that BSFC decreased steadily for both fuels as engine load increased. This trend can be explained by more efficient combustion at higher in-cylinder pressures and temperatures. In the present study, the BSFC of B20 decreased from 2.320 kg/kWh at the lowest load to 0.331 kg/kWh at full load, while for B20But10 it dropped from 2.417 to 0.348 kg/kWh. B20 exhibited lower BSFC values than B20But10 across all load ranges, which can be attributed to B20’s higher cetane number and calorific value, enabling more effective energy conversion^40^. However, ternary blends containing n-butanol consumed more fuel to produce the same power due to their lower energy content, resulting in increased BSFC. Similar findings have been reported in the literature, where alcohol-enriched blends exhibited higher BSFC due to reduced heating value and lower cetane number. ^103,104^Additionally, the lower cetane number of ternary blends extends ID, leading to greater fuel consumption during the premixed phase and contributing to higher BSFC^105^.

Effect of NP addition on BSFC showed a positive trend. Experimental data indicated that the lowest BSFC values were achieved with 75 ppm NP addition, reaching 0.295 kg/kWh for B20 and 0.333 kg/kWh for B20But10. The reduction in BSFC observed with NP addition may be associated with the ability of metal oxide NPs to promote a more homogeneous oxygen distribution within the fuel–air mixture and to facilitate oxidation-related processes inside the cylinder, as suggested in previous studies^52^. In addition, the high surface-to-volume ratio of NPs is reported to expand the effective reaction surface, potentially contributing to improved combustion efficiency^106^. Consistent with this interpretation, several studies have reported that nickel and nickel oxide NP additives can reduce BSFC. These effects have been discussed in the literature in terms of enhanced fuel atomization, increased catalytic activity, and shortened combustion duration^41,53,54^. In addition, previous studies have indicated that oxide NPs, owing to their oxygen-containing structure, may improve oxygen availability within the combustion zone and promote more complete oxidation processes^107^.

At the same time, it should be noted that BSFC is a system-level performance parameter, and small variations may also be influenced by measurement sensitivity or by slight changes in fuel physical properties such as density and viscosity, which can affect burette-based fuel consumption measurements. Therefore, the observed BSFC reduction should be interpreted as the combined outcome of fuel properties, combustion-related effects reported in the literature, and experimental measurement characteristics.

### Emissions performance

The evaluation of emission performance was carried out by focusing on the major pollutants typically produced in diesel engines, namely CO, NOx, HC, smoke and CO₂. These species were monitored across different operating conditions to understand how fuel composition and NP additives influence combustion completeness, mixture reactivity, and overall emission trends.

### Carbon monoxide emission

**Fig. 11:**
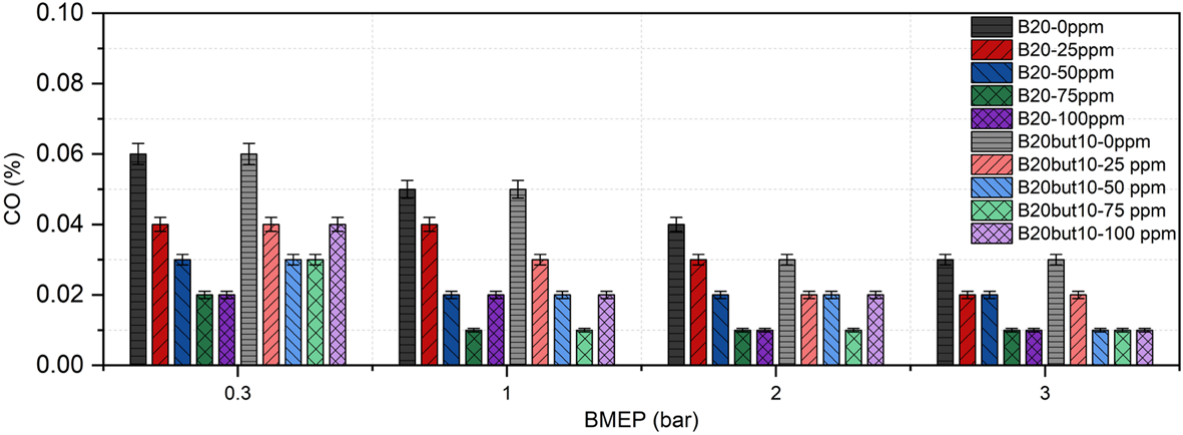
Variation of CO with NP and BMEP.

CO is an indicator of incomplete combustion and is sensitive to in-cylinder oxygen availability and flame temperature. Figure 11 shows the variation of CO emissions measured at different loads for B20 and B20But10 blends. The results demonstrate that CO decreases steadily for both fuels as engine load increases; this trend is consistent with higher temperatures and more complete oxidation at increased load^92^. Comparatively, B20But10 produced similar or lower CO emissions than B20 at most loads; this can be attributed to the higher oxygen content and lower C/H ratio of n-butanol, which facilitate the oxidation of CO to CO₂^108,109^, aligning with reports that alcohol-enriched blends reduce CO^110,111^. Moreover, studies have shown that CO emissions in ternary blends vary with the butanol fraction, with additional reductions observed as butanol content increases. This effect is explained by butanol’s lower density, which accelerates evaporation, reduces spray penetration, and enhances mixture homogeneity^112,113^.

The addition of NPs generally led to a gradual reduction in CO: with increasing NP concentration up to 75 ppm, CO values decreased significantly under low and medium loads. However, a slight rebound was observed in some conditions when increasing from 75 to 100 ppm, likely due to NP agglomeration and heat absorption at high doses, which can lower local flame temperature and weaken oxidation^114^. In contrast, at 2.0–3.0 bar BMEP, CO levels dropped to as low as 0.01–0.02% within the 50–75 ppm range, indicating that NPs improve oxygen distribution and accelerate the oxidation of carbon intermediates through surface catalytic effects^115^. Nickel and nickel oxide NPs, in particular, have been reported to promote CO to CO₂ conversion via catalytic action, supporting emission reduction^40,42,54^. Overall, load increase remains the dominant factor in suppressing CO, while n-butanol and NP additions provide further improvements; yet at 100 ppm, excessive dosing can cause marginal CO rebound under certain loads. This dual effect is also consistent with thermal explanations that lower flame temperatures at light loads limit CO oxidation^92^.

### Nitrogen oxides emissions

**Fig. 12:**
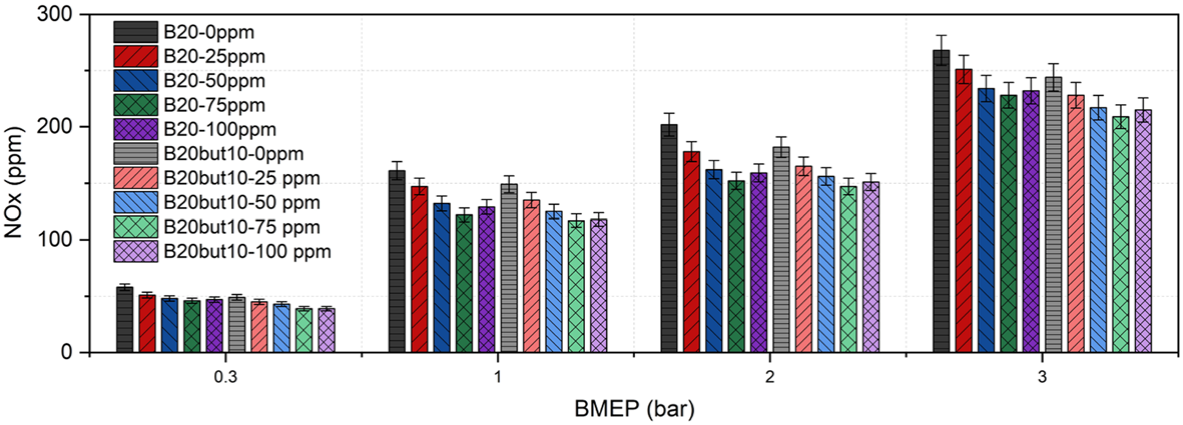
Variation of NOx with NP and BMEP.

NOx formation in diesel combustion is strongly related to peak temperature, equivalence ratio, and residence time at high temperature and is classically explained by the Zeldovich mechanism^116^. The curves presented in Fig. 12 show that NOx increases markedly with engine load for all fuels; however, both n-butanol addition and Ni₂O₃ supplementation significantly reduced absolute NOx levels. For example, at full load, NOx for neat B20 and B20But10 decreased from 268 and 244 ppm to 232 ppm and 215 ppm with 100 ppm NP addition. The relative reduction was even more pronounced at low load: for B20, NOx dropped from 58 to 47 ppm, and for B20But10 it decreased from 49 to 39 ppm. This systematic decrease can be attributed to the charge-cooling effect caused by the high latent heat of vaporization and lower heating value of n-butanol, which lowers peak flame temperatures; similar NOx suppression by butanol has been reported in the literature^18,117^. Comparable findings are also documented for ternary blends, where NOx reduction is linked to lower flame temperature^118,119^, and previous research emphasizes that butanol–diesel NOx behavior is sensitive to engine calibration and operating conditions^120^. Other important parameters affecting NOx formation include the oxygen content of the blends, fuel heating value, air–fuel ratio, and combustion duration^121–123^.

The effect of NP dosage shows a monotonic NOx reduction up to 75 ppm; at 100 ppm, a very slight rebound was observed for both fuels, likely due to increased heat absorption at higher loading, which can reshape peak temperature zones^108^. Up to 75 ppm, NP addition reduces NOx by shortening ID, moderating excessive heat release during the premixed phase, decreasing peak temperature and residence time, and improving micro-scale mixing and heat/mass transfer, thus limiting local hot spot formation. This temperature residence time-controlled NOx reduction observed with oxygenated and NP doped fuels is consistent with the literature^124^. Our results also agree with previous studies reporting NOx reduction through nickel and nickel oxide-based NPs^40,41^. Overall, the results confirm that engine load remains the dominant factor governing NOx formation, primarily through its influence on in-cylinder temperature and residence time. The addition of n-butanol provides the most pronounced NOx reduction, which is consistently attributed to its charge-cooling effect and lower flame temperature. In comparison, Ni₂O₃ addition plays a secondary but observable role, with its influence being more evident at low and medium load conditions, while only marginal changes are observed at high load. Therefore, the contribution of NPs should be regarded as a modulation of NOx formation rather than a primary controlling mechanism. Within this framework, 75 ppm appears to represent an effective dosage level, beyond which diminishing returns are observed.

### Hydrocarbons emissions

**Fig. 13:**
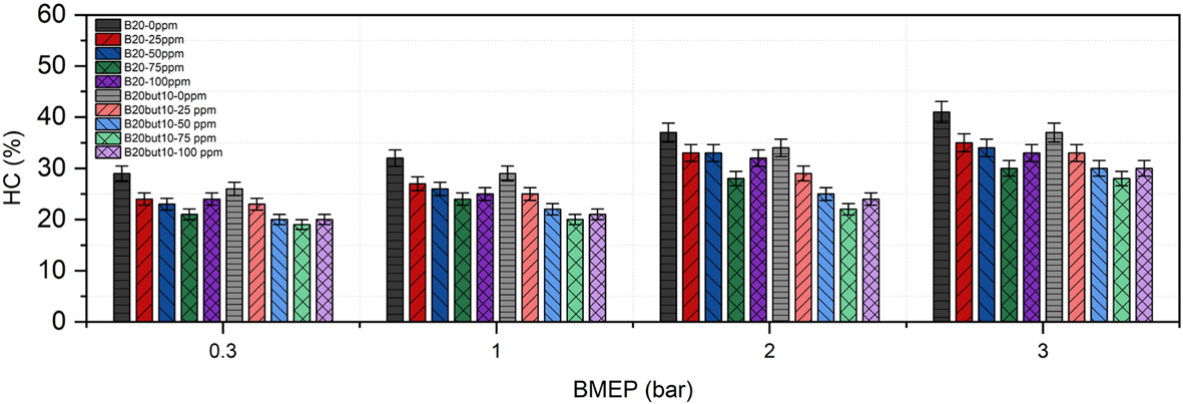
Variation of HC with NP and BMEP.

HC emissions, which result from incomplete oxidation during the combustion process, are closely related to the quality of the fuel air mixture and in-cylinder temperature conditions. As shown in Fig. 13, HC emissions increased with rising engine load for both B20 and B20But10 fuels. For the neat fuels at full load, HC levels were measured as 41 ppm for B20 and 37 ppm for B20But10, while these values decreased to 30 ppm and 28 ppm, respectively, with the addition of 75 ppm NP. This reduction can be explained by the oxygen content of butanol, which supports combustion and facilitates the oxidation of CO and HC^125,126^. However, the lower calorific value of biobutanol tends to reduce in-cylinder temperature, while higher compression ratios can help increase combustion temperature^127^. Additionally, studies have reported HC increases in ternary blends containing isobutanol due to its high latent heat of vaporization^111^, indicating that the effect of alcohol addition depends on the type and proportion of alcohol used.

The addition of NPs resulted in a clear reduction of HC emissions. This decrease is attributed to the catalytic activity of NPs, which accelerate the oxidation of unstable intermediate species^128^. However, at a concentration of 100 ppm, HC emissions slightly increased again, suggesting that excessive additive dosing may have a limited negative effect on combustion efficiency^129^. Overall, the findings are consistent with previous studies on nickel oxide, confirming the effectiveness of such additives in reducing HC emissions^41,54^.

### Smoke emissions

**Fig. 14:**
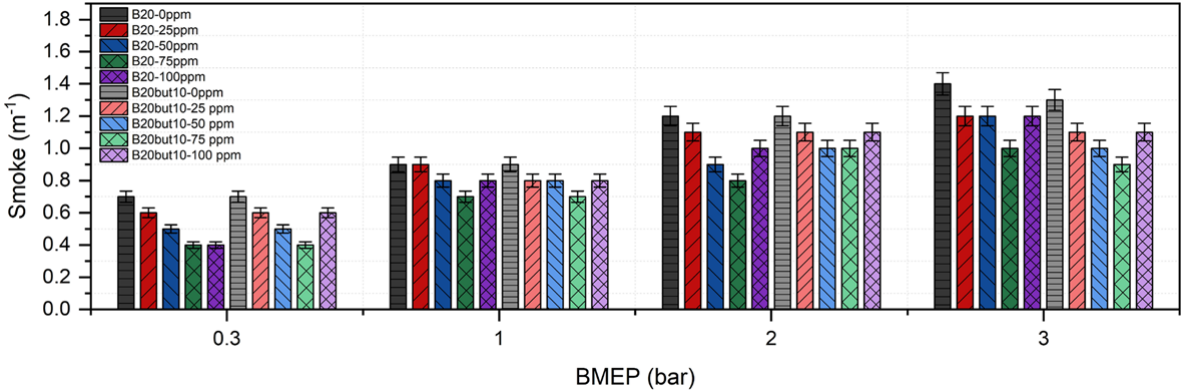
Variation of Smoke with NP and BMEP.

Smoke emissions in diesel engines are associated with the presence of carbon based particulates formed due to incomplete oxidation during the combustion process and are a critical parameter for both engine performance and environmental sustainability. As shown in Fig. 14, smoke intensity increased with engine load for both B20 and B20But10 fuel blends. At full load with neat fuels, smoke opacity was measured as 1.4 m⁻^1^ for B20 and 1.3 m^−1^ for B20But10; this difference can be explained by the lower carbon content of butanol and its oxygen bonds, which limit the formation of soot precursors^92,130^. The literature also reports that butanol can suppress soot formation by transporting oxygen into the pyrolysis region, thereby reducing particulate matter emissions^69,131^.

With NP addition, smoke emissions decreased more noticeably. At 75 ppm NP loading, smoke opacity dropped to 0.4 m^−1^ for both B20 and B20But10 at low load, representing a significant improvement compared to the neat fuels. This reduction can be attributed to the high surface to volume ratio of NPs, which enhances oxidation and improves combustion efficiency. However, at 100 ppm, a slight increase in smoke was observed under certain conditions (e.g., 0.3 bar BMEP with B20But10), suggesting that excessive additive concentration may cause agglomeration and heat absorption, weakening local reactivity. These results demonstrate the synergistic effect of butanol and NPs in suppressing soot formation. While biodiesel and butanol addition reduce smoke intensity by diluting aromatic precursors, the catalytic activity of NPs further supports oxidation and lowers emissions^94,132^. These findings are consistent with reported smoke reduction trends using nickel oxide and confirm the effectiveness of such additives in particulate control^53,57^. It should be noted that the smoke measurements reported in this study are based on exhaust opacity, which provides an indirect indication of particulate formation. Accordingly, the observed reductions in smoke opacity are interpreted as qualitative trends for comparative assessment of fuel and additive effects.

### Carbon dioxide emissions

**Fig. 15:**
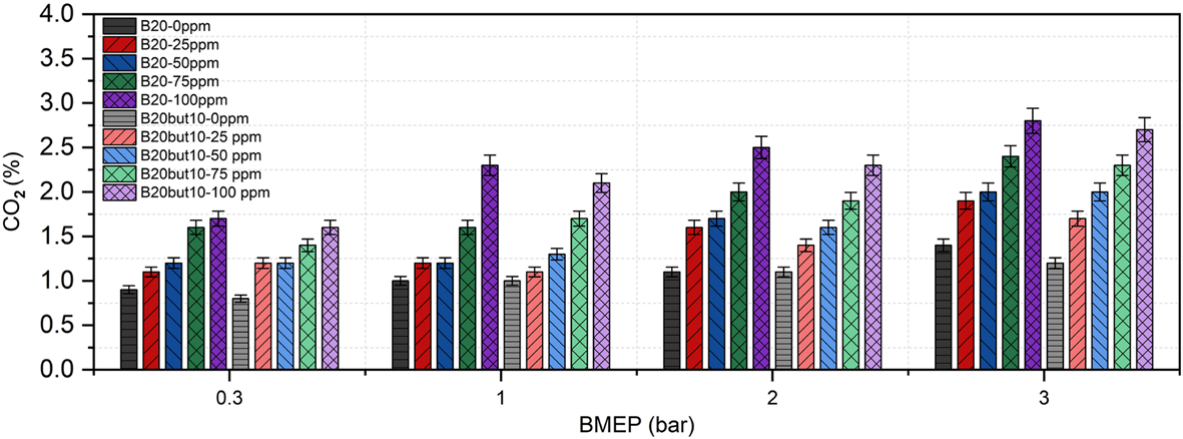
Variation of CO_2_ with NP and BMEP.

CO₂ emissions in internal combustion engines primarily result from the oxidation of carbon-containing species and are influenced by both fuel composition and operating conditions. As shown in Fig. 15, CO₂ emissions increased steadily with engine load for both B20 and B20But10 blends. At full load with neat fuels, CO₂ levels were measured at 1.4% for B20 and 1.2% for B20But10, which can be attributed to the lower C/H ratio and higher inherent oxygen content of butanol, limiting CO₂ formation^133^. Similar findings have been reported in the literature, where butanol shows lower Similar trends have been widely reported in the literature for butanol-containing fuels^110,134^.

With NP addition, CO₂ emissions increased progressively from 0 to 100 ppm, reaching 2.8% for B20 and 2.7% for B20But10 at full load. This increase may be associated with enhanced oxidation-related processes, including the conversion of CO to CO₂ ^106,135^, as discussed in previous studies on nickel and nickel oxide NPs^40,42^. However, it should be noted that CO₂ concentration alone cannot be regarded as a definitive indicator of combustion efficiency, as variations in air–fuel ratio, exhaust dilution, and measurement conditions may also influence the measured CO₂ levels. Therefore, the observed CO₂ trends are interpreted in conjunction with CO and HC emissions rather than as an isolated measure of combustion quality.

### RSM results and optimization

In this study, the influence of Ni₂O₃ ppm and engine load on the combustion, performance, and emission characteristics of B20 and B20But10 fuel blends was analyzed using the RSM. Regression models were established for each response variable, and their statistical significance was verified through analysis of variance (ANOVA). Table 9 presents the experimental results of the response variables obtained for different factor levels of NP concentration and BMEP under the RSM design.

**Table 9:** Design matrix.

RUN	NP (ppm)	BMEP (bar)	BTE (%)		BSFC (kg/kWh)		CO (%)		NOx (ppm)		HC (ppm)		Smoke (m)^−1^		CO_2_ (%)	
			B20	B20But10	B20	B20But10	B20	B20But10	B20	B20But10	B20	B20But10	B20	B20But10	B20	B20But10
1	0	0.3	3.52	3.32	2.320	2.417	0.06	0.06	58	49	29	26	0.7	0.7	0.9	0.8
2	0	1	9.11	9.10	0.910	0.960	0.05	0.05	161	149	32	29	0.9	0.9	1.0	1.0
3	0	2	17.42	17.39	0.479	0.489	0.04	0.03	202	182	37	34	1.2	1.2	1.1	1.1
4	0	3	23.96	23.89	0.331	0.348	0.03	0.03	268	244	41	37	1.4	1.3	1.4	1.2
5	25	0.3	3.61	3.36	2.294	2.393	0.04	0.04	51	45	24	23	0.6	0.6	1.1	1.2
6	25	1	9.36	9.32	0.908	0.955	0.04	0.03	147	135	27	25	0.9	0.8	1.2	1.1
7	25	2	17.64	17.22	0.468	0.486	0.03	0.02	178	165	33	29	1.1	1.1	1.6	1.4
8	25	3	24.06	23.95	0.323	0.348	0.02	0.02	251	228	35	33	1.2	1.1	1.9	1.7
9	50	0.3	3.73	3.41	2.282	2.381	0.03	0.03	48	43	23	20	0.5	0.5	1.2	1.2
10	50	1	9.49	9.45	0.899	0.951	0.02	0.02	132	125	26	22	0.8	0.8	1.2	1.3
11	50	2	17.88	17.46	0.462	0.478	0.02	0.02	162	156	33	25	0.9	1	1.7	1.6
12	50	3	24.22	24.68	0.319	0.340	0.02	0.01	234	217	34	30	1.2	1	2	2
13	75	0.3	3.83	3.71	2.125	2.284	0.02	0.03	46	39	21	19	0.4	0.4	1.6	1.4
14	75	1	9.60	9.63	0.886	0.939	0.01	0.01	122	117	24	20	0.7	0.7	1.6	1.7
15	75	2	17.98	17.82	0.450	0.451	0.01	0.01	152	147	28	22	0.8	1	2	1.9
16	75	3	24.85	24.94	0.295	0.333	0.01	0.01	228	209	30	28	1	0.9	2.4	2.3
17	100	0.3	3.79	3.50	2.193	2.359	0.02	0.04	47	39	24	20	0.4	0.6	1.7	1.6
18	100	1	9.58	9.52	0.890	0.949	0.02	0.02	129	118	25	21	0.8	0.8	2.3	2.1
19	100	2	17.89	17.50	0.455	0.471	0.01	0.02	159	151	32	24	1	1.1	2.5	2.3
20	100	3	24.89	24.75	0.309	0.339	0.01	0.01	232	215	33	30	1.2	1.1	2.8	2.7

The ANOVA analysis showed that both Ni₂O₃ dosage and engine load had a statistically significant effect on the majority of performance and emission parameters, with p-values < 0.05 (Table 10). In particular, BTE and CO₂ were found to be strongly dependent on BMEP (p = 0.000), while NP dosage was identified as a key factor influencing CO, HC, and smoke opacity. These results indicate that NP addition improves combustion kinetics. The consistently high R^2^ values (above 0.95), together with the associated confidence intervals reported in Table 11, indicate that the developed second-order polynomial regression models adequately describe the experimental responses. CCD was selected as it is specifically formulated to support second-order (quadratic) response surface models, which are well suited for capturing nonlinear behavior and interaction effects commonly observed in internal combustion engine responses. Preliminary analysis indicated that linear models were insufficient to represent the curvature associated with load and NP dosage effects, while higher-order models would introduce unnecessary complexity without physical justification. The derived equations incorporate not only the linear contributions of NP concentration and BMEP but also interaction terms, highlighting that the synergistic effects between engine load and additive dosage cannot be ignored. For example, the negative coefficient of NP concentration in the CO regression model confirms that increasing NP content reduces incomplete combustion products, whereas the positive quadratic NP term in the NOₓ related equations indicate that, beyond a certain threshold, the observed trends may be associated with enhanced thermal reactivity.

**Table 10:** p-values values resultant for each answer.

Factors	ANOVA										Coefficient of determination %					
	NP		BMEP		NP*NP		BMEP*BMEP		NP*BMEP		R^2^		R^2^ adjusted		R^2^ predicted	
	B20	B20But10	B20	B20But10	B20	B20But10	B20	B20But10	B20	B20But10	B20	B20But10	B20	B20But10	B20	B20But10
BTE	0.001	0.000	0.000	0.000	0.493	0.172	0.000	0.000	0.100	0.049	99.94	99.96	99.92	99.95	99.89	99.92
BSFC	0.582	0.744	0.000	0.000	0.935	0.891	0.000	0.000	0.682	0.831	96.70	97.01	95.52	95.95	93.79	94.43
CO	0.000	0.000	0.000	0.000	0.001	0.000	0.299	0.014	0.011	0.413	94.57	91.52	92.63	88.49	89.54	80.54
NOx	0.010	0.019	0.000	0.000	0.177	0.303	0.081	0.025	0.400	0.539	95.74	95.90	94.23	94.44	91.55	91.73
HC	0.000	0.000	0.000	0.000	0.000	0.000	0.109	0.026	0.288	0.294	94.91	97.98	93.09	97.26	90.36	94.39
Smoke	0.000	0.000	0.000	0.000	0.011	0.000	0.039	0.000	0.849	0.415	94.44	96.22	92.45	94.87	88.47	92.09
CO_2_	0.000	0.000	0.000	0.000	0.084	0.321	0.580	0.561	0.067	0.001	95.83	97.62	94.34	96.77	91.27	94.69

**Table 11:** 2nd order polynomial equations for responses.

B20	
BTE (%)	0.696 + 0.00534 NP + 9.206 BMEP − 0.000035 NP^2^ − 0.4855 BMEP^2^ + 0.00255 NP*BMEP
BSFC (kg/kWh)	2.811 − 0.00159 NP − 2.098 BMEP + 0.000003 NP^2^ + 0.4281 BMEP^2^ + 0.00043 NP*BMEP
CO (%)	0.06267 − 0.000775 NP − 0.01340 BMEP + 0.000003 NP^2^ + 0.00120 BMEP^2^ + 0.000071 NP *BMEP
NOx (ppm)	45.9 − 0.704 NP + 101.7 BMEP + 0.00529 NP^2^ − 9.35 BMEP^2^ − 0.093 NP*BMEP
HC (ppm)	26.41 − 0.1995 NP + 6.80 BMEP + 0.001486 NP^2^ − 0.683 BMEP^2^ − 0.00955 NP*BMEP
Smoke (m^-1^)	0.6133−0.00750 NP + 0.4041 BMEP + 0.000049 NP^2^ − 0.0508 BMEP^2^ + 0.000094 NP*BMEP
CO_2_ (%)	0.832 + 0.00395 NP + 0.151 BMEP + 0.000051 NP^2^ + 0.0209 BMEP^2^ + 0.001593 NP*BMEP

B20But10	
BTE (%)	0.554 + 0.00723 NP + 9.097 BMEP − 0.000058 NP^2^ − 0.4338 BMEP^2^ + 0.00252 NP*BMEP
BSFC (kg/kWh)	2.946 − 0.00121 NP − 2.225 BMEP + 0.000005 NP^2^ + 0.4589 BMEP^2^ + 0.00022 NP*BMEP
CO (%)	0.06434 − 0.000792 NP − 0.02222 BMEP + 0.000005 NP^2^ + 0.00382 BMEP^2^ + 0.000025 NP*BMEP
NOx (ppm)	35.1 − 0.528 NP + 102.5 BMEP + 0.00363 NP^2^ − 11.42 BMEP^2^- 0.0620 NP*BMEP
HC (ppm)	26.514 − 0.2138 NP + 1.944 BMEP + 0.001400 NP^2^ + 0.617 BMEP^2^ − 0.00583 NP*BMEP
Smoke (m^-1^)	0.5341 − 0.00743 NP + 0.5437 BMEP + 0.000063 NP^2^ − 0.0999 BMEP^2^ − 0.000288 NP *BMEP
CO_2_ (%)	0.8396 + 0.00539 NP + 0.0989 BMEP + 0.000020 NP^2^ + 0.0155 BMEP^2^ + 0.002359 NP*BMEP

Significant: 0.000 < *p* ≤ 0.05.

**Fig. 16:**
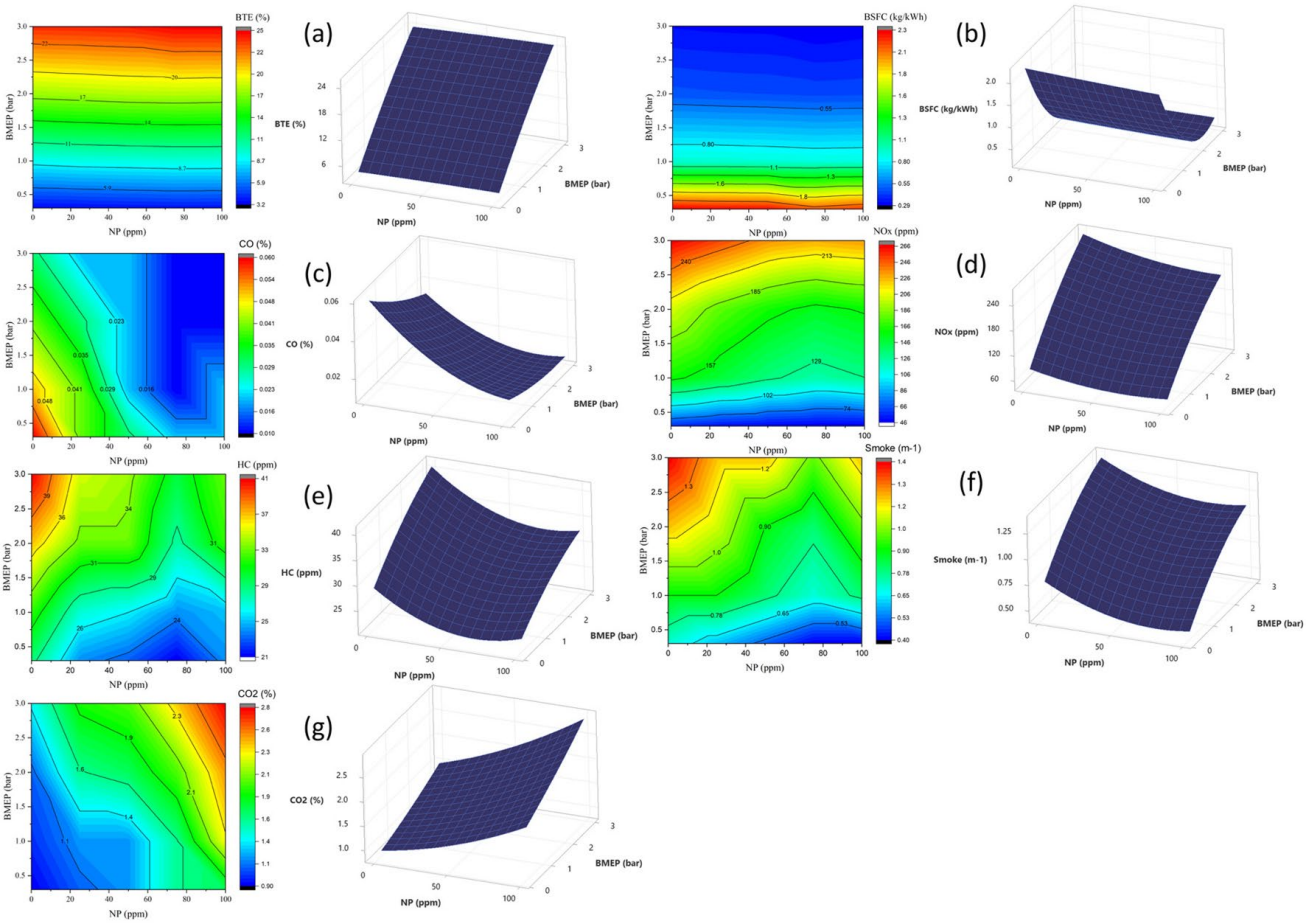
Variation of the models based on NP concentration and BMEP according to RSM: (**a**) BTE (**b**) BSFC (**c**) CO (**d**) NOx (**e**) HC (**f**) Smoke (**g**) CO₂.

The literature on direct ANOVA studies for nickel oxide additives is limited. However, studies with similar metal oxide NPs (e.g., ZnO and TiO₂) in biodiesel blends have reported that NPs are dominant factors in reducing CO and HC, while BMEP strongly influences BTE and NO_x_ formation^20,64^. Similarly, RSM based investigations using SiO₂ enhanced biodiesel systems have shown that NP concentration significantly affects smoke and CO₂, with nanomaterials catalytically promoting oxidation^136^. Figure 16 presents the distribution of response variables across the experimental design space, clearly showing that Ni₂O₃ addition systematically reduces CO and HC and improves BTE at high loads. These findings are consistent with biodiesel optimization studies, further confirming the strong predictive and optimization capability of RSM based models^137,138^.

Model adequacy was evaluated using standard RSM diagnostics, including coefficients of determination (R^2^, adjusted R^2^, and predicted R^2^), ANOVA results, and experimental–predicted validation outcomes. The close agreement between predicted and experimental values, together with low relative errors, indicates that the developed quadratic models adequately represent the system behavior within the studied design space.

According to Table 7 presented in the introduction, five different models were developed for the optimization of engine performance and emissions. To validate these models, verification tests summarized in Table 12 were conducted, and the obtained optimum results were experimentally reproduced. Thus, the predictive capability of each model was comparatively evaluated against experimental data. When the overall mean error percentages for both fuels were examined, the values were calculated as 6.10% for Model 1, 5.51% for Model 2, 3.48% for Model 3, 5.84% for Model 4, and 3.29% for Model 5. All these error values remain below the widely accepted error threshold of 7% reported in the literature^139^. Therefore, the developed models can be considered highly reliable. However, the highest average error was observed for CO emissions. This is mainly attributed to the ± 0.01 precision of the measuring instrument and the very low CO levels, which cause the relative error percentage to appear higher.

**Table 12:** Optimization and validation results for the models.

Model	NP (ppm)	BMEP (bar)	Composite desirability	Case	BTE (%)	BSFC (kg/kWh)	CO (%)	NOx (ppm)	HC (ppm)	Smoke (m^-1^)	CO_2_ (%)
B20 + NP											
1	63.636	1.363	0.640	O	12.76	0.69	0.017	135.62	26.89	0.79	1.67
				E	12.25	0.71	0.02	138.00	28.0	0.80	1.71
				Er %	-3.9	2.8	17.6	1.7	4.1	1.2	2.39
2	68.686	0.3	0.865	O	n/a	n/a	0.023	50.16	21.49	0.44	1.42
				E	n/a	n/a	0.02	51.0	23.0	0.41	1.37
				Er %	n/a	n/a	-13	1.6	7	-6.8	-3.5
3	98.989	3	0.981	O	24.88	0.36	n/a	n/a	n/a	n/a	n/a
				E	24.21	0.38	n/a	n/a	n/a	n/a	n/a
				Er %	-2.6	5.5	n/a	n/a	n/a	n/a	n/a
4	61.616	2.018	0.677	O	17.81	0.28	0.014	178.07	29.5	0.95	1.85
				E	18.15	0.27	0.02	182.0	28.0	0.91	1.82
				Er %	1.9	-3.5	42.8	2.2	-5	-4.2	-1.6
5	66.66	0.736	0.695	O	7.53	1.42	0.020	87.62	23.87	0.60	1.52
				E	7.89	1.51	0.02	89.1	24.0	0.57	1.48
				Er %	4.7	6.3	0.0	1.6	0.5	-5	-2.6
B20But10 + NP											
1	58.5859	1.390	0.636	O	12.79	0.70	0.015	132.04	22.21	0.85	1.58
				E	12.91	0.67	0.02	134.0	23.0	0.81	1.64
				Er %	0.9	-4.2	33.3	1.4	3.5	-4.7	3.8
2	61.6162	0.3	0.826	O	n/a	n/a	0.0302	44.887	19.185	0.46	1.32
				E	n/a	n/a	0.03	42.0	19.0	0.42	1.24
				Er %	n/a	n/a	-0.6	-6.4	-0.9	-8.7	-6.6
3	92.929	3	0.982	O	24.81	0.39	n/a	n/a	n/a	n/a	n/a
				E	24.27	0.38	n/a	n/a	n/a	n/a	n/a
				Er %	-2.1	-3	n/a	n/a	n/a	n/a	n/a
4	58.5859	1.963	0.674	O	17.25	0.31	0.010	166.75	24.31	0.96	1.74
				E	17.48	0.33	0.01	168.0	25.0	1.01	1.81
				Er %	1.3	6.4	0.0	0.7	2.8	5.2	4
5	59.596	0.818	0.682	O	8.05	1.38	0.022	89.69	20.46	0.67	1.43
				E	8.11	1.29	0.02	88.97	21.0	0.64	1.45
				Er %	0.7	-6.5	-9	-0.8	2.6	-4.4	1.4

O: Optimum value, E: Experimental value, Er: Error %.

**Fig. 17:**
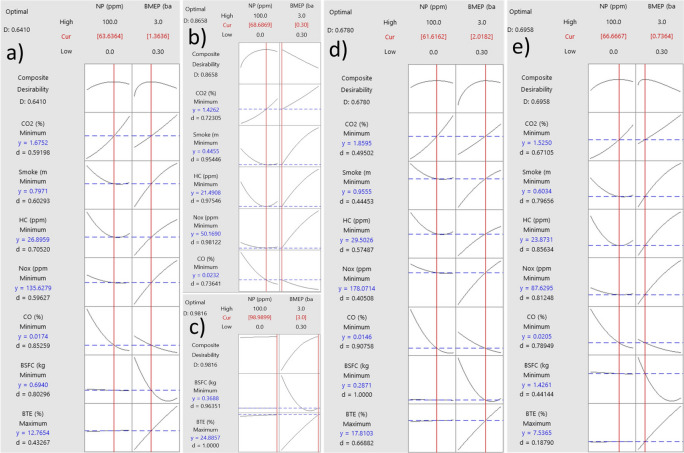
Optimization plot: B20 fuels for all cases. (**a**) Model-I, (**b**) Model-II, (**c**) Model-III, (**d**) Model-IV, (**e**) Model-V.

**Fig. 18:**
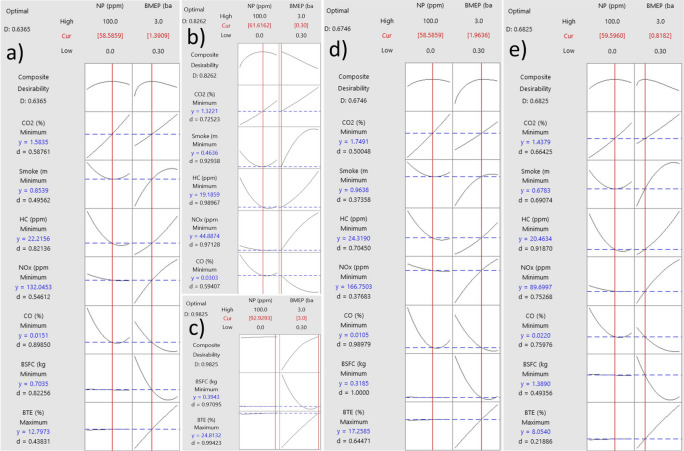
Optimization plot: B20But10 fuels for all cases. (**a**) Model-I, (**b**) Model-II, (**c**) Model-III, (**d**) Model-IV, (**e**) Model-V.

Figure 17 shows the optimization curves of the five models for B20 fuel, while Fig. 18 presents the corresponding curves for B20But10 blends. These models were analyzed based on maximum performance and minimum emissions criteria. Each model represents optimum conditions at different NP concentrations and engine loads. For instance, in Model 1, the optimum NP concentration was found to be 63.6 ppm for B20 and 58.5 ppm for B20But10; this difference may be related to B20’s higher carbon content requiring additional oxygen support during combustion. Model 2 represents purely emission focused conditions, with optimum points found at the lowest load level: 68.6 ppm for B20 and 61.6 ppm for B20But10. In the performance oriented Model 3, the highest NP concentrations were determined at full load, with 98.9 ppm for B20 and 92.9 ppm for B20But10. This can be associated with the increased heat release at higher loads, where higher NP levels enhance the oxidation process. Model 4, which emphasizes performance, showed moderate optimum NP levels (61.6 ppm for B20 and 58.5 ppm for B20But10) while maintaining relatively high BTE and BSFC values and thus providing a balanced effect. In the emission focused Model 5, optimum points for achieving low BTE and low emissions at light loads were identified as 66.6 ppm for B20 and 59.5 ppm for B20But10. The analysis indicates that the composite desirability values remain within acceptable limits and that all models produce statistically meaningful results^140^. Although direct optimization studies for nickel oxide are limited, similar RSM based research on metal oxide NPs such as ZnO and TiO₂ has reported comparable optimum concentration ranges^20,141^. These findings demonstrate that the optimum NP levels in Ni₂O₃ doped fuels vary depending on load and fuel composition, yet all the developed models provide robust and practical optimization outcomes.

### Economic analysis

An economic analysis was conducted to evaluate the cost aspect of using NP doped fuels in diesel engines. This analysis is important for assessing both the industrial-scale applicability of the fuel blends and their potential financial sustainability. According to data provided by Nanografi, the cost of the Ni₂O₃ used in this study is approximately $273 per 100 g^63^. For the preparation of the test fuels B20 and B20But10, raw material costs were taken into account. B20 fuel consists of 20% biodiesel (waste-derived methyl ester) and 80% diesel, with current market prices in Turkey of $1.33/L for biodiesel and $1.10/L for diesel^142^. The B20But10 blend additionally includes n-butanol, supplied by a local vendor at a cost of $1.77 per liter^143^. In this study, the combined fuel cost change was calculated using the BSFC change rate available in the literature^144^, and the formulation shown in Eq. (15) was applied^145^.

15 $$\frac{\Delta E}{{E}_{1}}x100=\frac{{E}_{2}-{E}_{1}}{E1}x100=\left[\frac{{X}_{i}+{\sum }_{2}^{n}{X}_{i}{R}_{i}}{{X}_{i}+{\sum }_{2}^{n}{X}_{i}{S}_{i}}\left(1+\frac{\Delta {B}_{e}}{{B}_{e}}\right)-1\right]x100$$

Here, the symbol E1 refers to the unit cost of the base fuel, while E2 denotes the cost of the NP doped fuel blend. The relative cost relationship between these two fuels is expressed as Ri, and Xi designates the volumetric proportion of the base fuel in the mixture. The parameter ΔBe/Be is used to represent the change in brake specific fuel consumption (BSFC), whereas Si indicates the density ratio of the tested fuels. In this framework, E1 and E2 are given in $ /L, Si in kg/m^3^, and Be in kg/kWh^64^.

**Fig. 19:**
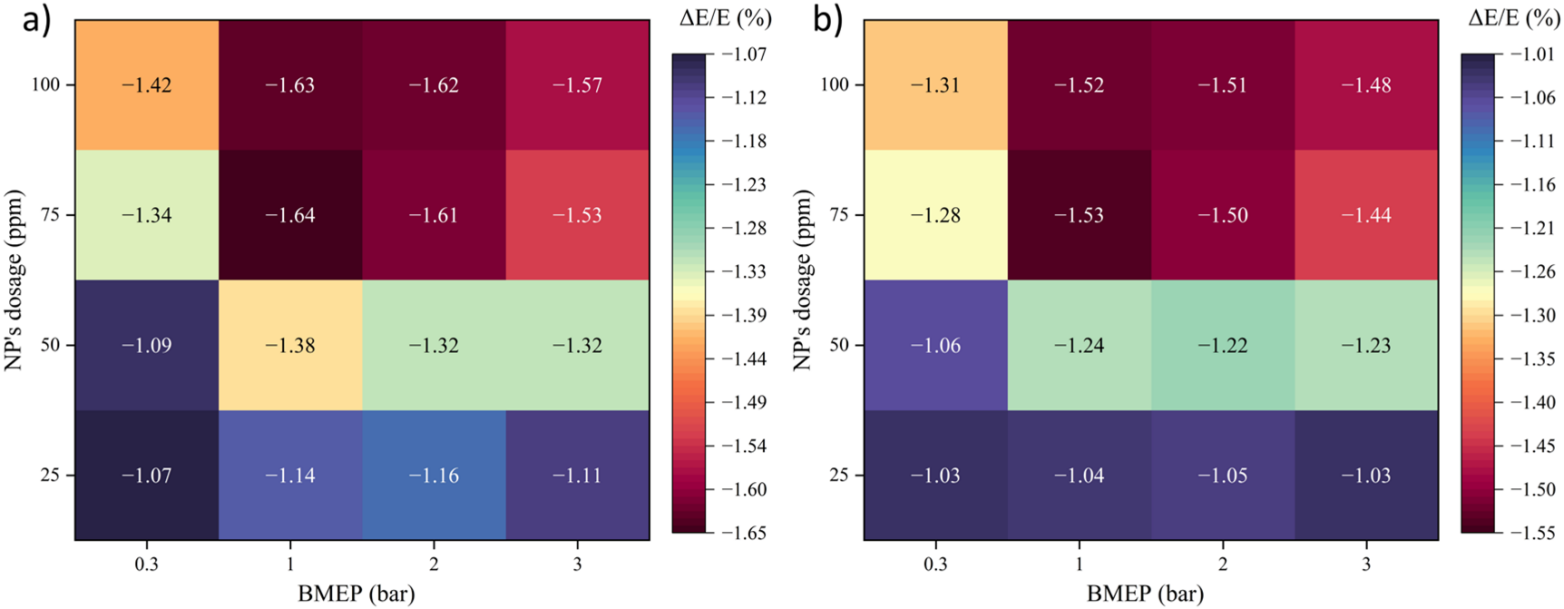
Economic analysis of test fuels (**a**) B20 + NP (**b**) B20But10 + NP.

The economic assessment revealed that the incorporation of NP into the fuel blends consistently reduced overall operating costs when compared with the baseline condition. The extent of this reduction was primarily influenced by the NP dosage, while variations in engine load did not show a systematic relationship with economic gain. The analysis indicated that higher NP concentrations generally resulted in greater savings, and the most favorable outcome was recorded at 75 ppm. Under this condition, fuel costs decreased by 16.4% for B20 and by 15.3% for B20But10, highlighting the cost-effectiveness of Ni₂O₃ addition in both blends. The observed trends and reductions are shown in Fig. 19 across different NP concentrations and load conditions.

## Conclusion

In this study, Ni₂O₃ was added at different concentrations (25, 50, 75, and 100 ppm) to B20 and B20But10 fuel blends, and engine performance, combustion, and emission behaviors were comprehensively investigated under four different loads (0.3, 1.0, 2.0, and 3.0 bar BMEP) using a single-cylinder, four-stroke, water-cooled, direct injection (DI) diesel engine, while statistical and economic analyses were also conducted. The results obtained can be summarized as follows:

1. In NP doped B20 and B20But10 blends, increases were observed in density, kinematic viscosity, and cetane index, whereas decreases were recorded in calorific value and flash point. For B20 fuel within the 0–100 ppm range, density increased by 0.18%, viscosity by 5.13%, and cetane index by 3.8%, while calorific value and flash point decreased by 5.9 and 4.4%, respectively. For B20But10, density increased by 0.24%, viscosity by 6.4%, and cetane index by 5.3%, while calorific value and flash point decreased by 5.7% and 10.7% over the same range.
2. Combustion analyses suggested potential improvements in atomization and oxidation behavior with Ni₂O₃ addition. At full load, the CP increased from 53.03 bar to 55.86 bar for B20 and from 52.21 bar to 55.45 bar for B20But10; the maximum HRR reached 29.45 J/°CA and 30.02 J/°CA, respectively. This indicates that NP addition strengthened premixed combustion.
3. Regarding engine performance, at full load BTE increased from 23.96 to 24.89% for B20 and from 23.89 to 24.94% for B20But10, while BSFC decreased from 0.331 to 0.309 kg/kWh and from 0.348 to 0.333 kg/kWh, respectively.
4. NP addition generally yielded favorable results for emissions. CO emissions at 100 ppm decreased from 0.03% to 0.01% for both blends. HC emissions decreased by 13–28%, smoke opacity by 8–43%, and NOₓ by 12–21%. In contrast, CO₂ emissions increased by approximately 2–9% due to more complete combustion. Despite minor fluctuations at low load and high dosage, overall trends suggested improved combustion efficiency and emission behavior with Ni₂O₃ addition. Additionally, the n-butanol-containing blend achieved greater reductions in CO, HC, and smoke compared to B20 due to its additional oxygen content, while the increase in CO₂ remained more limited compared to B20.
5. The second-order regression models developed using RSM provided high reliability (R^2^ = 90.9–99.9). Error analyses remained within the range of 2–6%, and composite desirability values were found to be between 0.63 and 0.98. For the five models created, Model 1 with equal importance levels achieved optimum points of 63.3–58.5 ppm at 1.3 BMEP for B20 and B20But10, respectively; the emission-focused Model 2 achieved 68.6–61.6 ppm at 0.3 BMEP; and the performance-focused Model 3 reached 98.9–92.9 ppm at full load. These results demonstrate that Ni₂O₃ addition can be flexibly optimized according to the engine’s primary targets (performance or emission).
6. Adding Ni₂O₃ to the fuel blends was found to be economically feasible, as it improved energy conversion efficiency and reduced fuel consumption. The highest cost savings were obtained at 75 ppm, with fuel costs reduced by 16.4% for B20 and 15.3% for B20But10.

As a result, Ni₂O₃ enhanced the reactivity and combustion homogeneity of biodiesel and n-butanol blends, improving performance, reducing emissions, and providing economic advantages through lower fuel consumption. This study demonstrates that Ni₂O₃ is a promising additive for clean diesel technologies and can make significant contributions to sustainable energy and emission reduction goals.

## Limitations and future research directions

- The present study primarily focuses on the short-term effects of Ni₂O₃ NP addition on combustion, performance, and emission characteristics. Potential long-term durability aspects, including injector wear, abrasive interactions, ash accumulation, and lubricant stability, were not experimentally investigated and therefore represent limitations of the present work. Although metal oxide nanoparticles have been reported to pose wear-related risks under prolonged operation, these effects are generally associated with higher additive loadings and extended operating durations^146,147^. In the present study, the relatively low nanoparticle dosages employed (25–100 ppm) together with the short-term experimental conditions indicate that the likelihood of such effects becoming immediately significant is low. Nevertheless, comprehensive long-term engine durability tests and tribological analyses are required to fully evaluate these potential effects.
- The environmental fate of nickel-containing exhaust particulates was not investigated within the scope of the present study and therefore represents an additional limitation. Previous studies have reported that the high reactivity and bioavailability of nickel-based metal oxide NPs may pose potential risks to environmental systems and human health under specific exposure conditions and prolonged release scenarios^148,149^. However, the present study employs low NP dosages (25–100 ppm) under short-duration engine operating conditions, which are not intended to represent long-term environmental exposure. Accordingly, for the safe and responsible application of Ni₂O₃ NPs in fuel-related applications, appropriate exposure limits should be established and life-cycle-based environmental impact assessments, including emission, dispersion, and post-combustion fate, should be comprehensively evaluated in future studies.
- Injection timing was kept constant in the present study to enable a controlled comparison of fuel effects; therefore, fuel-specific injection optimization was not considered and represents a limitation. Future studies incorporating optimized injection strategies are recommended to further elucidate the influence of fuel properties on combustion phasing, efficiency, and emissions.
- Smoke emissions were evaluated based on exhaust opacity, which provides an indirect indication of particulate formation. Advanced particulate characterization techniques, such as soot mass concentration or particle size distribution analysis, were beyond the scope of this work. Future research employing detailed particulate measurements would provide deeper insight into combustion–emission interactions and particulate formation mechanisms.
- At higher concentrations (≥ 100 ppm), Ni₂O₃ exhibited a tendency to agglomerate and settle over time, which may restrict long-term fuel storage stability. Therefore, clarifying the optimal additive level is important for ensuring both fuel stability and engine operability.
- For NO_x_ emissions, the limited reductions or occasional increases observed under certain operating conditions suggest that additional measures to improve in-cylinder temperature control may be beneficial. The long-term effects of such strategies on engine durability and maintenance requirements remain insufficiently understood.
- Future research should focus on optimizing NP concentration, enhancing fuel stability, evaluating injector deposit formation, assessing compatibility with exhaust after-treatment systems, and performing comprehensive life-cycle cost analyses.

## Data Availability

Data can be obtained from the corresponding author upon reasonable request.

## Abbreviations

ANOVA: Analysis of variance
B: Biodiesel
BMEP: Brake mean effective pressure
BP: Brake power
BSFC: Brake specific fuel consumption
BTE: Brake thermal efficiency
But: Butanol
CA, θ: Crank angle
CCD: Central composite design
CHRR: Cumulative heat release rate
CP: In-cylinder pressure
CO: Carbon monoxide
CO_2_: Carbon dioxide
CV: Calorific value
D: Diesel
E: Ethanol
EGT: Exhaust gas temperature
FTIR: Fourier-transform infrared spectroscopy
HC: Hydrocarbons
HRR: Heat release rate
ID: Ignition delay
K: Kelvin
MINITAB: Data analysis software
Ni_2_O_3_: Nickel (III) oxide
NOx: Nitrogen oxide
NP: Nanoparticle
PM: Particulate matter
PRR: Pressure rise rate
RSM: Response surface methodology
R^2^: Coefficient of determination
SEM: Scanning electron microscopy
SOC: Start of combustion
SOI: Start of injection
XRD: X-ray diffraction
